# Evaluating how clear the questions being investigated in randomised trials are: systematic review of estimands

**DOI:** 10.1136/bmj-2022-070146

**Published:** 2022-08-23

**Authors:** Suzie Cro, Brennan C Kahan, Sunita Rehal, Anca Chis Ster, James R Carpenter, Ian R White, Victoria R Cornelius

**Affiliations:** 1 Imperial Clinical Trials Unit, School of Public Health, Imperial College London, London, UK; 2 Medical Research Council Clinical Trials Unit at University College London, London, UK; 3 GlaxoSmithKline, London, UK; 4 Kings College London, London, UK; 5 London School of Hygiene and Tropical Medicine, London, UK; Correspondence to: S Cro s.cro@imperial.ac.uk (or @Suzie_cro on Twitter)

## Abstract

**Objectives:**

To evaluate how often the precise research question being addressed about an intervention (the estimand) is stated or can be determined from reported methods, and to identify what types of questions are being investigated in phase 2-4 randomised trials.

**Design:**

Systematic review of the clarity of research questions being investigated in randomised trials in 2020 in six leading general medical journals.

**Data source:**

PubMed search in February 2021.

**Eligibility criteria for selecting studies:**

Phase 2-4 randomised trials, with no restrictions on medical conditions or interventions. Cluster randomised, crossover, non-inferiority, and equivalence trials were excluded.

**Main outcome measures:**

Number of trials that stated the precise primary question being addressed about an intervention (ie, the primary estimand), or for which the primary estimand could be determined unambiguously from the reported methods using statistical knowledge. Strategies used to handle post-randomisation events that affect the interpretation or existence of patient outcomes, such as intervention discontinuations or uses of additional drug treatments (known as intercurrent events), and the corresponding types of questions being investigated.

**Results:**

255 eligible randomised trials were identified. No trials clearly stated all the attributes of the estimand. In 117 (46%) of 255 trials, the primary estimand could be determined from the reported methods. Intercurrent events were reported in 242 (95%) of 255 trials; but the handling of these could only be determined in 125 (49%) of 255 trials. Most trials that provided this information considered the occurrence of intercurrent events as irrelevant in the calculation of the treatment effect and assessed the effect of the intervention regardless (96/125, 77%)—that is, they used a treatment policy strategy. Four (4%) of 99 trials with treatment non-adherence owing to adverse events estimated the treatment effect in a hypothetical setting (ie, the effect as if participants continued treatment despite adverse events), and 19 (79%) of 24 trials where some patients died estimated the treatment effect in a hypothetical setting (ie, the effect as if participants did not die).

**Conclusions:**

The precise research question being investigated in most trials is unclear, mainly because of a lack of clarity on the approach to handling intercurrent events. Clear reporting of estimands is necessary in trial reports so that all stakeholders, including clinicians, patients and policy makers, can make fully informed decisions about medical interventions.

**Systematic review registration:**

PROSPERO CRD42021238053.

## Introduction

The results of randomised controlled trials are used in policy making and clinical practise to make decisions about which medical interventions to use. However, informed decision making requires an understanding of the precise question being investigated in a trial, because different questions can lead to different conclusions about the usefulness of an intervention.^1^ ^2^ ^3^ ^4^ ^5^ ^6^ ^7^ ^8^ ^9^ For example, a trial in type 2 diabetes^10^ compared a once weekly insulin regimen with a once daily regimen on the change from baseline in glycated haemoglobin, and asked two different questions. Firstly, what was the treatment effect if all participants had hypothetically adhered to the treatment regimens and not received ancillary treatment (hypothetical effect); and secondly, what was the treatment effect regardless of the amount of randomised treatment or ancillary treatment received (treatment policy effect). The hypothetical effect was twice as large as the treatment policy effect (mean difference −0.18 percentage points (95% confidence interval −0.38 to 0.02, P=0.08) *v* −0.09 (−0.29 to 0.20, P=0.35)).^10^ Therefore, depending on which treatment effect was considered most relevant in decision making, conclusions can differ substantially.

However, the specific questions that trials investigate are not always clear, and often stems from ambiguity in how events after randomisation (eg, intervention discontinuation or use of rescue therapy; termed as intercurrent events) are handled in the definition of the treatment effect. In some cases, the relevant information is omitted, or expert statistical knowledge might be required to decipher this from reported methods. For example, a placebo controlled trial in atopic dermatitis reported baricitinib in combination with topical steroids significantly reduced impairment in daily activities.^11^ All randomised participants were included in the analysis in their randomised group, so readers using the results to inform decision making might assume that the trial addressed the intervention’s effect if adopted into routine practice. However, on close inspection of the statistical methods, the trial assessed the intervention effect in the hypothetical situation where participants who stopped treatment had instead continued and rescue therapy was denied. This interpretation is because investigators set outcomes recorded after discontinuation and receipt of rescue therapy to missing, and then used a statistical model that implicitly imputed what the participant’s outcome would have been had they not discontinued treatment or received rescue therapy (table 1).

**Table 1:** Deciphering the research question being investigated in example trial^11^*

Research question (estimand†) attribute	Definition of attribute	Method of statistical analysis used in example trial* (estimator)	Inferable attribute from reported statistical analysis (estimator) in atopic dermatitis trial‡
Population	Population of patients targeted by clinical question (ie, who the treatment effect is for)	Analysis included all randomised participants	Adult patients with atopic dermatitis, as defined by the trial inclusion and exclusion criteria
Treatment conditions	Treatment strategies being compared	All randomised participants were included in a repeated measure analysis using a mixed model. Data after rescue therapy or treatment discontinuation was set missing (see handling of intercurrent events); model included fixed effects for treatment and treatment-by-visit and data up to time of first rescue therapy or treatment discontinuation	Baricitinib (4 mg) or placebo daily plus topical corticosteroids continued through to week 16, and without rescue therapy
Outcome variable	Endpoint or measure collected for each patient	The model outcome was the change in Work Productivity and Activity Impairment-AD absenteeism domain score between baseline and 16 weeks	Change in Work Productivity and Activity Impairment-AD absenteeism domain score from baseline at 16 weeks
Handling of intercurrent events	Specification of how to account for intercurrent events§	Data after treatment discontinuation and rescue therapy use were set to missing; model implicitly imputed missing data using the data from participants who did not discontinue or require rescue therapy	Treatment discontinuation was handled using a hypothetical strategy, as if all treatment was adhered to (even if discontinued due to adverse event); rescue therapy was handled using a hypothetical strategy, as if no rescue therapy was received
Population level summary measure for outcome	Targeted summary measure for outcome variable, used to compare treatment conditions (eg, mean difference, risk ratio, odds ratio)	Model estimated mean change from baseline and included a covariate for treatment group	Mean treatment group difference in outcome
**Description of overall research question (estimand) inferred from reported methods (estimator)**			
What is the mean change from baseline in Work Productivity and Activity Impairment-AD absenteeism domain score at 16 weeks for baricitinib 4 mg daily plus topical corticosteroids versus placebo plus topical corticosteroids, continued through to week 16, and without rescue therapy for adults with atopic dermatitis, as defined by trial’s inclusion and exclusion criteria			

* Example trial is placebo controlled trial investigating baricitinib plus topical corticosteroids versus placebo plus tropical corticosteroids in atopic dermatitis (Wollenberg A, Nakahara T, Maari C, et al).^11^

† Table 2 provides definitions and another example of an estimand, estimator, and estimate.

‡ Inferred from the statistical methods (ie, the estimator) for Work Productivity and Activity Impairment-AD outcomes; absenteeism domain and 4 mg dose used as example.

§ Strategies for handling intercurrent events summarised in ICH E9(R1) are described in table 2.

To tackle such issues and avoid trial results being misinterpreted, new international trial regulatory guidance (ICH E9(R1), the International Council for Harmonisation of Technical Requirements for Pharmaceuticals for Human Use (ICH) E9(R1) addendum on Estimands and Sensitivity Analyses in Clinical Trials, November 2019^12^) has called for trials to precisely define the clinical questions being assessed by specifying estimands. An estimand is a precise description of the treatment effect that a trial is aiming to find out (ie, the question to be answered). It expresses what the numerical result (the estimate) represents including with respect to intercurrent events. It is entirely separate to the statistical methods (the estimator), which specifies how the trial will compute the result. An example estimand and strategies for handling intercurrent events are outlined in table 2. 

**Table 2:** Definitions of estimands, estimators, and estimates, using an example trial^13^*

Definition	Example*
**Estimand**	
An estimand is a precise description of the question being investigated in a clinical trial. It defines what treatment effect researchers want (or demand) to find out and includes five attributes (targeted population, treatment conditions, outcome variable, handling of intercurrent events, and population level summary measure for the outcome).† To handle intercurrent events, several strategies exist: Treatment policy—the occurrence of an intercurrent event is irrelevant or does not matter, and the treatment effect, regardless of (or despite) an intercurrent events occurrence, is targeted Hypothetical—the treatment effect in a particular hypothetical scenario is targeted (eg, if an intercurrent event does not occur) Composite—the occurrence of an intercurrent event is included in the definition of the outcome variable (eg, intercurrent event is assigned a particular value of the outcome variable) While on treatment—the treatment effect is of interest only before the occurrence of an intercurrent event Principal stratum—the treatment effect in a subset of the population whose intercurrent event status would be identical, irrespective of treatment group is targeted (eg, the subgroup of the population who would always adhere)	Estimand description: What is the mean difference in the change from baseline in PPPASI score at eight weeks, in patients with a confirmed diagnosis of palmoplantar pustulosis (meeting the trial eligibility criteria) treated with anakinra compared with placebo, regardless of treatment discontinuation for any reason, initiation of rescue, or prohibited or other topical therapy. Attributes of estimand: Population—patients with confirmed diagnosis of palmoplantar pustulosis meeting trial eligibility criteria Treatment conditions—anakinra compared to placebo regardless of treatment discontinuation for any reason, initiation of rescue, or prohibited or other topical therapy Outcome variable—change from baseline in PPPASI score at eight weeks Handling of intercurrent events—treatment policy applied for study treatment discontinuation, use of rescue therapy, prohibited therapy, and other topical therapy Population level summary measure—mean difference
**Estimator **	
An estimator is the statistical machinery or method that researchers use to get from what they want to know (the estimand) to knowing the answer (the estimate); it is how you get to what you want to know.	Analysis was based on the intention-to-treat principle and included all randomised participants with at least one follow-up in the group to which they were randomised, regardless of subsequent treatment received. A linear mixed effect model estimated the mean difference in PPPASI at eight weeks between groups, adjusted for baseline and using all observed data (including data observed after treatment discontinuation, rescue therapy, prohibited therapy, or other topical therapy).
**Estimate**	
An estimate is a numerical result. The estimand describes what the numerical result represents.	–1.65, 95% confidence interval –4.77 to 1.47; P=0.30

PPPASI=Palmoplantar pustulosis area and severity score.

* Example trial looks at effect of anakinra on Palmoplantar pustulosis (Cro S, Cornelius VR, Pink AE, et al).^13^

† First three attributes might be recognisable from the PICO framework (patient/population, intervention, comparison, and outcomes),^14^ but alone are insufficient, and require careful consideration because they are typically affected by intercurrent events.

Current trial reporting guidelines (CONSORT^15^) were established before the introduction of ICH E9(R1) and do not require trialists to specify estimands. As this area of focus is new, and while medicine regulators worldwide are adopting ICH E9(R1) guidelines, we aimed to determine current practise and establish whether the reporting of estimands in trial reports is necessary to fully understand the questions being investigated in clinical trials. In our study, we reviewed published randomised trials with the specific objectives of evaluating how often the precise question being assessed in a trial was stated or could be determined from the reported methods using statistical knowledge, and to identify what questions are being investigated. 

## Methods

The protocol for this systematic review is in the supplementary material and is registered on PROSPERO.^16^

### Search strategy

We examined randomised controlled trials published in the year 2020 in six high impact general medical journals: *Annals of Internal Medicine*, *The BMJ*, *Journal of the American Medical Association* (*JAMA*), *The Lancet*, *New England Journal of Medicine* (*NEJM*), and *PLOS Medicine*. We searched in February 2021 for articles in PubMed with a publication type of “randomised controlled trial,” or including the keyword “random*” in the title or abstract, or categorised with the MeSH term “random allocation.” The full search strategy is in appendix 1 in the supplement.

### Eligibility

Phase 2-4 randomised trials in humans were eligible for inclusion, with no restrictions on medical conditions or on interventions or comparators. Cluster randomised, crossover, non-inferiority, and equivalence trials were excluded since estimands and statistical issues within estimation might be different for these trials. Other exclusions included pilot or feasibility studies, phase 1 studies, non-randomised studies, secondary analyses of previously published trials, a primary outcome of cost effectiveness, more than one trial reported in the article (including meta-analysis and systematic reviews), interim analyses, or letters or commentaries.

Title and abstract screening of search results for eligibility was performed by one author (SC). Full texts of articles were then assessed independently by two statistical reviewers to confirm eligibility and extract data (SC, BCK, SR, or ACS).

### Data extraction

Data were extracted onto a pre-piloted standardised data extraction form (see supplement). Disagreements were resolved by discussion, or a third statistical reviewer where necessary. Where the trial publication referred to supplementary material (excluding a protocol or statistical analysis plan), the extractor referred to these documents. Extracted data included: trial characteristics, occurrence of intercurrent events, and whether the primary estimand was described for the trial’s primary objective or if information on the statistical methods (estimator) or other reported methods could be used to determine unambiguously what the estimand was using statistical knowledge. We also extracted whether supplementary estimands, defined as treatment effects that handled intercurrent events in a different way for the primary outcome, were described. Data on estimand specification in protocol and statistical analysis plans were extracted separately where these documents were available in supplementary material or referenced within the main article and publicly available.

### Outcomes

For each trial’s primary estimand, two statistical reviewers independently assessed whether each of the five estimand attributes (table 1) was explicitly stated, not explicitly stated but unambiguously inferable, or not inferable using similar methods to those used in a recent review of estimands in protocols.^17^ If an estimand was described as primary we used this as the primary estimand; if none or multiple estimands were listed as primary, we used the main analysis of the primary outcome to determine the primary estimand. If no analysis approach was described as the main or primary analysis, we used the first analysis approach listed in the statistical methods section for the trials’ primary outcome.

For intercurrent events, we first determined which of eight event categories referenced within the ICH E9(R1) addendum were relevant to the trial (see appendix 2 in the supplement), and then extracted data on the handling for the relevant events. In line with ICH E9(R1)^12^ and as described in table 2, strategies for dealing with intercurrent events were categorised as treatment policy, hypothetical, while-on-treatment, composite, or principal stratum. The overall strategy for handing intercurrent events was recorded as “stated” if the handling of all occurring intercurrent events was explicitly stated; “inferable” if the handling of all occurring events could be deduced from information in the publication or were stated; and “not inferable if one or more occurring intercurrent events handling was not inferable. Where non-treatment policy strategies were used—for example, the treatment effect for all patients with diagnosis of interest if all individuals adhered to medication, the statistical methods used for estimation were extracted.

Attributes were considered as “stated” if these were explicitly described as part of an estimand definition or if listed as part of the trial objective. For example, if the article included a description of the estimand and stated that the targeted population was all patients meeting the trial inclusion or exclusion criteria, this attribute would be classed as stated.

Attributes were “inferable” if they were not stated as part of an estimand definition or trial objective, but could be unambiguously deduced based on the statistical methods (estimator) or other reported methods using statistical knowledge. For example, if the article stated that the analysis population was all randomised participants and the analytical approach also targeted an effect for the full population, then the population attribute would be inferred as all patients meeting the trial inclusion or exclusion criteria. If an intention-to-treat analysis was specified and data collection continued after the occurrence of intercurrent events, the treatment condition could be inferred as the offer of treatment, and a treatment policy strategy could be inferred for handling non-terminal intercurrent events. If the statistical methods stated the type of summary measure that would be estimated (eg, a mean difference), or it was clear from the analysis model what the estimated measure was (eg, linear regression model), then the population level summary measure could be inferred (eg, targeted a mean difference).

Alternatively, if it was not clear how to reconstruct the estimand attribute or if more than one estimand was consistent with the reported information, attributes were “not inferable.” For example, if the analysis population excluded participants who did not receive all treatment, such as a per protocol analysis, it was unclear whether a principal stratum population of patients who would receive all treatment was of interest or whether the entire trial population meeting inclusion or exclusion criteria under a hypothetical strategy was of interest. Since both interpretations are consistent with the presented information, the population was set as “not inferable.” In this scenario, we also set intercurrent events handling to “not inferable” because it was not clear whether this corresponded to a hypothetical or principal stratum strategy.

The overall estimand was considered as “stated” if all five attributes were clearly stated, “inferable” if the five attributes were a mix of stated and inferable, or “not inferable” if one or more attributes was not inferable. We also assessed how well supplementary estimands that handled intercurrent events in a different manner to the primary estimand for the trial’s primary outcome were described. Where protocol or statistical analysis plans were available, we separately assessed whether these documents stated any estimands.

### Statistical methods

Outcomes were summarised descriptively using frequencies and percentages. We performed two prespecified subgroup analyses, which summarised outcomes separately by trial sponsor (pharmaceutical or for-profit (eg, medical device companies) *v* academic/not-for-profit) and self-defined pragmatic trial (pragmatic *v* not pragmatic). Analyses were performed using Stata version 15.

### Patient and public involvement

Patients and the public were involved in the initiation and interpretation of this study. The occurrence and potential impacts of post-randomisation events, such as treatment non-adherence, in clinical trials were described to members of the public (n=9, aged 20-60 years, mixed sex and ethnic groups) at a people’s research cafe run by the NIHR Imperial Patient Experience Research Centre. They supported the importance of researchers appropriately taking into account such events in clinical trials and the conduct of this research to understand how this is done. As the results of this study emerged, we reviewed selected outcomes with the public advisory panel for the HEALTHY STATS research project (NIHR300593). The public advisory panel aims to improve information reported from clinical trials for patients and health care practitioners: it includes five public partners aged 20-70 years of mixed ethnic groups and sex. The group were surprised that the type of question investigated in a clinical trial is not always clear: they highlighted the need for the specific trial question to be reported alongside the numerical results to ensure clarity. 

## Results

### Search results and trial characteristics

The search identified 753 articles, of which 255 were eligible randomised controlled trials (eFig 1). Most trials (175, 69%) had a drug intervention; 162 (64%) had an academic or not-for-profit sponsor; 93 (36%) had a pharmaceutical or for-profit sponsor; and the median sample size was 402 participants. Further trial characteristics are summarised in eTable 1 in the supplement.

### Primary estimand

**Fig 1:**
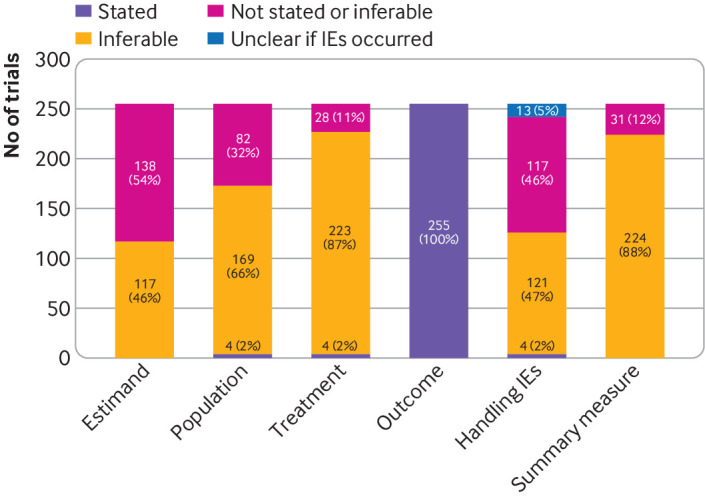
Description of primary estimand reported in 255 eligible randomised controlled trials, by estimand attribute. Table 1 provides definitions of estimand attributes. IE=intercurrent events (eg, intervention discontinuation or use of rescue therapy)

No articles completely stated the primary estimand (fig 1). Four (2%) trials attempted to explicitly state the estimand, but each of them omitted one or more attribute (table 3). The treatment effect investigated in the trial could be determined from the reported methods for 117 (46%) trials, as all estimand attributes were inferable. We were unable to determine the target population in 82 (32%) trials, treatment condition in 28 (11%) trials, handling of intercurrent events in 117 (46%) trials, and the population level summary measure in 31 (12%) trials. Reasons why attributes were stated, inferable, or could not be determined are presented in eTables 2-3 in the supplement.

**Table 3:** Attribute details of primary estimands in eligible randomised controlled trials

Attribute detail of primary estimand (No of trials)	No of trials	Proportion (%) of trials where attribute is stated or inferable	Proportion (%) of total No of trials (n=255)
**Population (n=173)**			
All eligible participants	169	98	66
All eligible participants with a prespecified baseline characteristic	4	2	2
Not stated or inferable	82	NA	32
**Treatment condition (n=227) **			
Treatment offer regardless of any intercurrent event (treatment policy)	175	77	69
Treatment offer, given a specified surgery or procedure was received	3	1	1
Treatment offer, with no use of another specified treatment	1	0	0
Initiating treatment	36	16	14
Initiating treatment but with no rescue therapy	1	0	0
Receiving all treatment	8	4	3
Receiving all treatment but with no use of another specified treatment	2	1	1
Receiving a specific amount of treatment	1	0	0
Not stated or inferable	28	NA	11
**Strategy for handling intercurrent events (n=125)**			
Composite	5	4	2
Hypothetical	2	2	1
Treatment policy	96	76	38
Treatment policy and composite	7*	6	3
Treatment policy and hypothetical	15†	12	6
Not stated or inferable	117	NA	46
**If unclear whether any intercurrent events occurred or were relevant, can the strategy for handling any potential intercurrent events be inferred (n=13)**			
No	2	15	1
Treatment policy inferable (intention-to-treat analysis)	11	85	4
**Population level summary measure (n=224) **			
Binary outcome:			
Odds ratio	17	8	7
Risk difference	26	12	10
Risk ratio	24	11	9
1−risk ratio (vaccine efficacy)	2	1	1
Continuous outcome:			
Standardised mean difference	2	1	1
Geometric mean ratio	1	0	0
Mean difference	63	28	25
Mean difference for area under curve	1	0	0
Median difference	5	2	2
Median ratio	1	0	0
Count/rate:			
Incidence rate ratio	7	3	3
Survival/time-to-event:			
1−risk ratio (vaccine efficacy)	1	0	0
Hazard ratio	66	29	26
Ordinal:			
Proportional odds ratio	8	4	3
Not stated or inferable	31	NA	12

NA=not applicable; intercurrent events=events that occur after randomised and affect the interpretation or existence of patient outcomes (eg, intervention discontinuation or use of rescue therapy); treatment policy=planned treatment course but not necessarily received. Percentages might not add up to 100% owing to rounding.

* One strategy (n=4 trials) classed as inferable from the reported statistical methods was considered difficult to infer and consisted of an analysis of a time-to-event outcome following the intention-to-treat principle where participants who died were censored at last observation day; this analytical approach includes deaths by infinite time to events^18^ (see eTable 9).

† Two strategies (n=3 trials) classed as inferable from the reported statistical methods were considered difficult to infer and included the following: one trial that used a joint model for a continuous outcome (change in the estimated glomerular filtration rate from baseline modelled using linear mixed model) and time to trial discontinuation due to death or end stage kidney disease before end of 104 week follow-up (Weibull parametric survival model), and that followed the intention-to-treat principle; and two trials that used marginal models on competing risk for recurrent events to handle terminal competing events, and that followed the intention-to-treat principle. Consensus was reached that these two approaches inferred a hypothetical estimand strategy with respect to the competing terminal intercurrent events since the outcome was not collected after the occurrence of the competing terminal intercurrent events, and the resulting treatment effect is estimated conditionally on a patient specific frailty (random effect) that models the correlation between the outcome and occurrence of the competing terminal intercurrent events^19^ (see eTable 9).

### Intercurrent events

**Fig 2:**
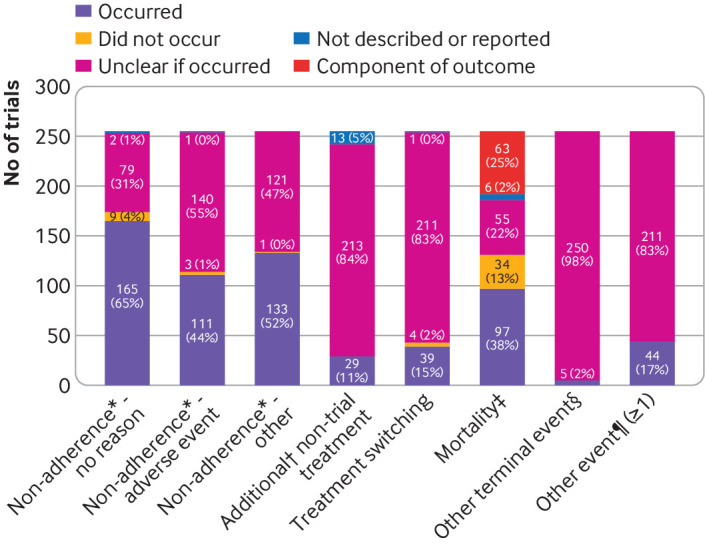
Intercurrent events occurring in eligible randomised controlled trials (n=255). Unclear if occurred=intercurrent event described in the introduction or methods but no frequency data reported, therefore was potentially an intercurrent event but not possible to ascertain whether actually occurred in the trial. *Non-adherence=treatment non-adherence or discontinuation for the given reason. †Additional=not part of usual care (eg, rescue or prohibited treatment). ‡An intercurrent event is defined as an event which occurs after randomisation and effects the existence of interpretation of trial outcomes; where death was the primary trial outcome, by definition this excludes the possibility of death being an intercurrent event. §Other terminal events observed include graft failure, termination of pregnancy, miscarriage or medical termination of pregnancy <20 weeks, pregnancy loss <22 weeks, cancelled surgery (outcome pain use within first 24 hours post-surgery). ¶Other intercurrent events are listed in eTable 4; 33 (13%) trials had one other intercurrent event, nine (4%) trials had two other intercurrent events, and two (1%) trials had three other intercurrent events

Two hundred and forty two (95%) trials reported at least one intercurrent event that could affect the interpretation of outcome data (table 4 and fig 2). We could determine how intercurrent events were handled in 125 (49%) trials (n=4 stated strategy, n=121=inferable). Where stated or inferable, most trials used a treatment policy strategy for handling all relevant intercurrent events (96/125, 77%), meaning that they considered the outcome regardless of any intercurrent events. Hypothetical or composite strategies were used to handle at least one type of intercurrent event for 17/125 (14%) and 12/125 (10%) trials. Statistical methods used for estimation of non-treatment policy strategies are summarised in table 4. Four (4%) of 99 trials with treatment discontinuation due to an adverse event estimated the treatment effect in a hypothetical setting, if participants continued to take treatment despite adverse events, and 19 (79%) of 24 trials with deaths considered a hypothetical setting, if participants did not die. Strategies for other intercurrent events are shown in eTable 5-6 in the supplement. Subgroup analyses by sponsor type and pragmatic trial design did not reveal any notable differences (eTables 7-8 in the supplement).

**Table 4:** Statistical methods used for handling intercurrent events in eligible randomised controlled trials, by strategy (excluding treatment policy strategies)

Statistical method	No of primary estimands (n=29 estimands, n=29 trials)	No of supplementary estimands (n=35 estimands, n=28 trials)
**Composite strategies**		
Participant with IE treated as non-responder or treatment failure	6*	—
Participant with IE treated given poor outcome (change in outcome=0)	1	—
Participant with terminal IE (death) given worst outcome (score 0 on continuous scale)	1	—
Participant with deaths at any time point during follow-up censored at last observation day—time-to-event analysis (ie, those who died treated as an infinite time to event)	4	4
Competing risk model—Fine and Gray (ie, those individuals who died are treated as an infinite time to event)	—	9
Joint rank model including all survival events (ranked participants by time to death and then by change in outcome; ranked score then analysed as outcome)	—	1
Imputed as having been administered antibiotics (primary count outcome) for the remainder of time after death (a composite strategy)	—	1§
**Hypothetical strategies **		
Time-to-event data censored at the time of IE (time-to-event analysis)	10	4
Data after IE set to missing—mixed model	2	1¶
Data after IE set to missing—multiple imputation	—	4**
Data after IE set to missing—inverse probability weighting	—	2
Data after IE not collected as does not exist (terminal IE)—multiple imputation	2†‡	—
Data after IE not collected as does not exist (terminal IE)—competing risk (terminal IE) marginal model for recurrent events	2	—
Data after IE not collected as does not exist (terminal IE)—joint model for outcome and time to terminal IE	1	—
Data after IE not collected as does not exist (terminal IE)—inverse probability weighting	—	1
Data after IE not collected as does not exist (terminal IE)—imputation with lower value than previous score for participants that died	—	1
**Principal stratum strategies**		
Complier average causal effect (CACE) analysis (implemented using latent growth mixture model (n=1), instrumental variable regression (n=3), structural mean model (n=1), or stated a CACE model (n=1))	—	6††
Instrumental variables analysis that estimated the change in treatment effect per unit change in compliance	—	1

IE=intercurrent event.

* One trial stated assumption of an unfavourable value after intercurrent event.

† One trial stated that “Group B IC [intercurrent] events were assumed to follow a hypothetical scenario, in which iGFR [measured glomerular filtration rate] values after developing ESKD [end stage kidney disease] take on biologically plausible values that are not confounded by the IC event, i.e., by ESKD treatments such as dialysis or kidney transplant. Group C IC events were assumed to conform to a hypothetical scenario, in which post-IC iGFR values have a similar distribution to other non-ESKD subjects with similar characteristics and pre-IC iGFR values.”^20^

‡ One trial used referenced based multiple imputation to impute data after death; the other trial used a combination of missing-at-random (MAR) and missing-not-at-random (MNAR) multiple imputation for two different intercurrent events (MAR multiple imputation after death, and MNAR multiple imputation after diagnosis or treatment for end stage kidney disease).

§ Trial assumed “any resident who died due to infection will have been taking antibiotics on all missing diary days.”^21^ eTable 9 summarises analysis methods for which the inferring of the strategy for handling intercurrent events was difficult.

¶ Trial assumed that “withdrawn subjects, had they completed the trial, would not have behaved differently than completing subjects from the same treatment arm with the same baseline characteristics and change in body weight at time of withdrawal.”^22^

** Two trials used MAR multiple imputation, one trial used delta based multiple imputation with a tipping point analysis, and one trial used placebo based MNAR multiple imputation post IE.

†† Three trials stated the following assumptions: “that a participant’s treatment was protocol compliant or non-compliant, when compliance status was indeterminable. (2 CACE [complier average causal effect] analyses done). (CACE1 assumes compliance where compliance status is indeterminable, CACE2 assumes non-compliance where compliance status is indeterminable)”^23^; “that members of the control group have the same probability of non-compliance as members of the intervention group; that members of the intervention group have the same probability of contamination as members of the control group; and that being offered the treatment has no effect on the outcome”^24^; “the treatment has no effect in the non-compliant subset.”^25^

### Supplementary estimands

One hundred and twelve (44%) trials used at least one supplementary estimand that handled intercurrent events in a different manner to the primary estimand. Sixty three (56%) of these trials incorrectly indicated that the supplementary estimand would deal with the same question as the primary estimand (ie, by mislabelling the supplementary estimand as a sensitivity analysis), which could cause confusion if results differ. No supplementary estimands were fully stated. One or more supplementary estimand was inferable for 28 (25%) trials including supplementary analysis (eTable 10-11 in the supplement). The handling of intercurrent events for one or more supplementary estimand was stated or inferable for 34 (30%) of 112 trials, including 28 (82%) using at least one non-treatment policy approach. Other strategies used included hypothetical, composite, or principal stratum (see table 4 for statistical methods).

### Use of estimands in protocols and statistical analysis plans

For 231 (91%) 255 articles, a protocol or statistical analysis plan was available (198 supplementary material, 25 published, and eight on references website). Of these 231 trials, 18 (8%) used the term “estimand” in the protocol or statistical analysis plan, including 16 with a pharmaceutical or for-profit sponsor and two with an academic or not-for-profit sponsor. The primary estimand was defined and fully stated by trial authors for four (2%) trials and partially stated for 10 trials (4%) within the trial protocol or statistical analysis plan; the remaining four trials that used the “estimand” term did not actually define what the estimand was (eTable 12 in the supplement). The handling of at least one intercurrent event was stated for 14 trials. Comparison with the results article revealed that eight trials had intercurrent events occurring that had not been planned for within the estimand. The stated estimand attributes are summarised in eTables 13-15 in the supplement and show variability on what is being stated.

## Discussion

### Principal findings

For over half of the 255 trials in this study, the precise primary question being investigated in the trial could not be determined unambiguously from the trial publication. While post-randomisation intercurrent events that could affect interpretation of outcome data occurred in most trials (95%; eg, treatment non-adherence, use of rescue therapy or mortality), the lack of clarity in handling these was the main driver for uncertainty on the question being answered. Where the primary trial question could be unambiguously determined from the reported methods (46%), most trials considered the occurrence of such intercurrent events as irrelevant in the calculation of the treatment effect and looked at the effect of the intervention regardless (ie, they used a treatment policy strategy). Other trials alternatively looked at how the intervention under study performed in a hypothetical scenario (eg, if the intercurrent event did not occur) or used a composite approach to incorporate the occurrence of intercurrent events into the outcome. Because the answers to different questions can result in different views on treatment benefit, the trial question should be explicitly stated by including statement of the estimand to avoid misinterpretations.

### Strengths and limitations

We conducted a systemic search and followed a pre-registered protocol.^16^ A standardised and piloted form was used for data extraction, which was conducted in duplicate by experienced trial statisticians using similar methods used in a recent review of estimands in protocols.^17^

We only included articles from six high impact medical journals, all of which follow the CONSORT statement, so we may have found worse reporting around estimands had we included a wider range of journals that did not endorse CONSORT. However, previous research indicates that despite journal endorsement of reporting guidelines, reporting might still be suboptimal.^15^ ^26^ ^27^

For many trials, we were able to infer what the estimand was from the study methods. However, we had no way of knowing whether the question addressed by the methods corresponded with what the trial investigators wanted to know. Without clear specification of estimands, it is impossible to assess whether the conducted analysis was appropriate for the originally targeted question.

### Research in context

Despite the growing recognition of the importance of precisely defining the research question,^12^ ^28^ ^29^ ^30^ ^31^ ^32^ ^33^ ^34^ ^35^ ^36^ ^37^ this review shows that the use of estimands is still far from routine. Many of the included trials would have been designed before the ICH E9(R1) publication (draft published 2017,^37^ final publication 2019^12^), and adoption by regulatory agencies worldwide (ICH E9(R1) adopted by ICH members Switzerland and Singapore in November 2019, Europe and Canada in July 2020, Taiwan in February 2021, US in May 2021, China in January 2022; and currently in the process of adoption for Korea, Japan, and Brazil^38^). Therefore, few trials explicitly stated the primary estimand. Of note, many of these trials were reported in line with the CONSORT guidelines, and so were reported according to best practice at the time. However, CONSORT guidelines were published before ICH E9(R1) and do not require trials to specify estimands, only the trial objective and how the numerical result (the estimate) was calculated (the statistical method or estimator). We have shown that this limited requirement does not always enable one to unambiguously infer what question was investigated. Therefore, we recommend that in any future update, estimands be explicitly incorporated into the CONSORT statement.^15^ More recent guidelines for the contents of statistical analysis plans in early phase trials include specification of estimands.^39^

However, this review found that some trials defined their estimands in their protocol, which differs from a recent review of protocols published in October 2020 in *Trials* and *BMJ Open*, where no protocols explicitly defined the estimand, and estimands could be inferred in only 26% of protocols.^17^ Use of estimands in this review might have been higher as we considered the six leading general medical journals and non-published protocols submitted as supplementary material.

### Implications

Researchers should describe estimands in trial reports so that the precise research questions being addressed for medical interventions can be understood by all. While 46% of primary estimands could be inferred from the reported methods by our statistical reviewers, inferability is likely to be lower for typical clinical readers and other non-methodologists, including patients reading trial results. Specifying estimands has a clear benefit here: it breaks down the details behind technical language, enabling transparent interpretation for all without the need for statistical knowledge or input.

Although certain aspects of the estimand seem new and potentially difficult to specify (such as intercurrent events), in practice, the events encapsulated by this label (eg, treatment discontinuation) have been around decades and have always required thought. Rather than leaving readers to guess how these have been handled (which might turn out to be incorrect), it is useful to clarify what research question has been used with respect to such events. Trialists should carefully consider plausible intercurrent events for their individual trial setting. Examples of intercurrent events are provided in ICH E9(R1) and we have summarised those identified in this review in figure 2 and eTable 4. Certain types of trials will have other specific events to consider.

No strategies for handling intercurrent events (table 2) can be universally recommended. These will be context specific, depending on the study objectives and stakeholders and require a multidisciplinary discussion during initial trial planning to establish. ICH E9(R1) indicates how the disease under study, clinical context (eg, availability of other treatments), administration of treatment, goal of treatment (eg, symptom control or cure), and experimental situation (eg, whether it differs to that anticipated in clinical practise) should be considered when establishing the strategy.

In this review, the intercurrent event whose handling was most often not inferable was mortality—generally, the treatment policy strategy does not apply to terminal intercurrent events such as death, because patient outcomes do not exist after death.^12^ When participants who die are excluded from trial analysis, it is not clear whether the intention is to estimate a hypothetical treatment effect if deaths did not occur, or the treatment effect for the subset of patients who would survive only. Because the resulting estimates are likely to differ, the handling of mortality needs to be explicitly stated where relevant.

In general, when considering a hypothetical strategy, researchers should ensure that the hypothetical scenario is clinical relevant and justified. We found that some trials assessed the treatment effect in a hypothetical setting if participants continued to take treatment despite adverse events; it is debatable how clinically relevant such a scenario is. The principal stratum strategy affects who the trial result applies to (the population attribute). Thus, the generalisability of results should be given careful consideration to ensure relevance for clinical practise in the light of the strategy used. Finally, while-on-treatment and composite strategies both affect the definition of the outcome variable, so the impact on interpretability of the trial must be thought through when such strategies are used.

Although we found some evidence of estimands being specified in the protocol, it is not realistic to expect that readers of the article, including practicing clinicians and patients, will seek this out. Moreover, these documents are not always made available with results.^40^ ^41^ For the four trials that fully stated their primary estimand in the protocol, none of the main results articles or their supplementary appendices mentioned that the estimand could be found there. We believe the estimand, including all five attributes, should be clearly stated in the results article or in a supplementary appendix and referenced in the main article, to avoid misinterpretation. Reviewers of trial results articles should have this issue in mind to allow fully informed decisions to be made by all trial stakeholders. CONSORT guidelines should be updated to mandate reporting of estimands. These actions will ensure that there is no room for misinterpretation of results, and is in line with ICH E9(R1) recommendations that are now adopted by regulatory agencies. Use of estimands will help future reviewers evaluate whether appropriate methods have been used.

### Conclusion

Understanding the research question being investigated in a trial is essential for informed decision making, but most often it is not clear precisely what the question is. Use of estimands can help clarify the precise study question. Trialists should explicitly describe estimands in trial reports, thereby allowing all stakeholders (including clinicians, patients and policy makers) to make fully informed decisions about medical interventions.

**What is already known on this topic**

In randomised trials, events after randomisation (such as intervention discontinuation or use of additional medications, termed intercurrent events) create ambiguity about how to define and interpret the treatment effect
To deal with such issues and avoid misinterpretations of results, new international trial guidelines (ICH E9(R1)) have called for a precise description of the research question the trial aims to address (ie, the estimand) to be provided
How often the precise trial question can be understood from the main results article and what questions are being used in trials is currently unclear

**What this study adds**

For most trials, the specific research question being investigated could not be understood from reported methods
Clear reporting of estimands is necessary in trial reports to avoid misinterpretation and to understand precisely what has been estimated, which is required for informed decision making around medical interventions in policy and medical practice

## Data Availability

The datasets used during the current study are available from the corresponding author at s.cro@imperial.ac.uk fon reasonable request.

## Web extra

Extra material supplied by authors

Web appendix: Supplementary materials

## Notes

Contributors: SC conceived of this study. SC, BCK, JRC, IRW, and VRC developed the protocol and data extraction forms. SC, BCK, SR, and ACS extracted the data. SC analysed the data and wrote the first draft of the manuscript. All authors revised the manuscript. All authors read and approved the final manuscript. All authors had full access to all of the data (including statistical reports and tables) in the study and can take responsibility for the integrity of the data and the accuracy of the data analysis. SC is the study guarantor. The corresponding author attests that all listed authors meet authorship criteria and that no others meeting the criteria have been omitted.

The lead author (the manuscript’s guarantor) affirms that the manuscript is an honest, accurate, and transparent account of the study being reported; that no important aspects of the study have been omitted; and that any discrepancies from the study as originally planned (and, if relevant, registered) have been explained.

Dissemination to participants and related patient and public communities: We intend to disseminate the importance of understanding the trial question to a public audience to facilitate the understanding of trial results, and will seek further patient and public involvement from the HEALTHY STATS public advisory panel in the development of an appropriate method of dissemination. Dissemination to the wider clinical trial community will be undertaken via presentations at relevant conferences and seminars.

Provenance and peer review: Not commissioned; externally peer reviewed.
