# Supplementary material for: Evaluating how clear the questions being investigated in randomised trials are: systematic review of estimands
Source: BMJ. 2022 Aug 23;378:e070146. doi: 10.1136/bmj-2022-070146 (PMC9396446; doi:10.1136/bmj-2022-070146)
Supplement: Supplementary file 1 — Web appendix: Supplementary materials [file cros070146.ww.pdf]

# Supplementary File for: Evaluating how clear the questions being investigated in randomised trials are: systematic review of estimands

## Table of Contents


Appendix 1: Search Strategy ........ 2

Appendix 2: Classification of Intercurrent events ........ 3

Appendix 3: Flow chart of search results ........ 3

eFigure 1: Flow chart of search results ........ 3

Appendix 4: Additional Results ........ 4

eTable 1 – Characteristics of eligible trials (total number of trials 255) ........ 4

eTable 2 – Reasons why population, treatment condition and population level summary measure attributes were stated, inferable or non-inferable ........ 5

eTable 3 – Reasons why handling of intercurrent events were stated, inferable or non-inferable... 6

eTable 4 – Listing of other intercurrent events by trial (44 Trials, 11 other IEs) ........ 9

eTable 5 - The handling of intercurrent events by type of event ........ 10

eTable 6 - Statements provided by trial authors for handling intercurrent events ........ 11

eTable 7 - Subgroup analysis by sponsor (academic/not for profit versus pharmaceutical/for profit) ........ 13

eTable 8 - Subgroup analysis by pragmatic trial design ........ 16

eTable 9 – Analysis where inferring of the strategy for handling intercurrent events was difficult 19

eTable 10 - Supplementary IE estimand details including strategies and analysis (n=154 supplementary estimands in 112 trials) ........ 21

eTable 11 - Summary of estimand use in Protocols or SAPs ........ 23

eTable 12 - Primary Estimand stated/partially stated in Protocol or SAP (n=14) ........ 25

eTable 13 Estimand attributes stated by the trial authors in protocol/SAP populations: ........ 26

eTable 14 - Primary Estimands stated/partially stated in Protocol (n=9) ........ 27

eTable 15 - Primary estimands stated/partially stated in SAP (n=13) ........ 28

Appendix 6 – List of reviewed articles (n=255) ........ 29

References ........ 46

## Appendix 1: Search Strategy

**PubMed:**

1.“randomized controlled trial"[Publication Type] OR "random allocation"[MeSH Major Topic] OR random*[Title/Abstract]

2."BMJ"[Journal] OR "JAMA"[Journal] OR "Lancet (London, England)"[Journal] OR "The New England journal of medicine"[Journal] OR "PLoS medicine"[Journal] OR "Annals of internal medicine"[Journal]

3."2020/01/01"[Date - Publication] : "2020/12/31"[Date - Publication]

4. 1 AND 2 AND 3

## Appendix 2: Classification of Intercurrent events

Intercurrent events were classified as; (1) Treatment non-adherence/discontinuations where no reason was specified, (2) Treatment non-adherence/discontinuation due to an adverse event, (3) Treatment non-adherence/discontinuation due to a specified reason excluding adverse event, (4) Use of additional non-trial treatment that is not part of usual care (i.e. rescue/prohibited therapy), (5) Treatment switching (to another randomised treatment), (6) Mortality, (7) Other terminal events excluding mortality when measurement becomes impossible and not part of outcome (e.g. ankle amputation in a trial assessing ankle function) and (8) Other. These categories were selected as events referenced within the ICH-E9 R1 addendum [1].

## Appendix 3: Flow chart of search results

### eFigure 1: Flow chart of search results

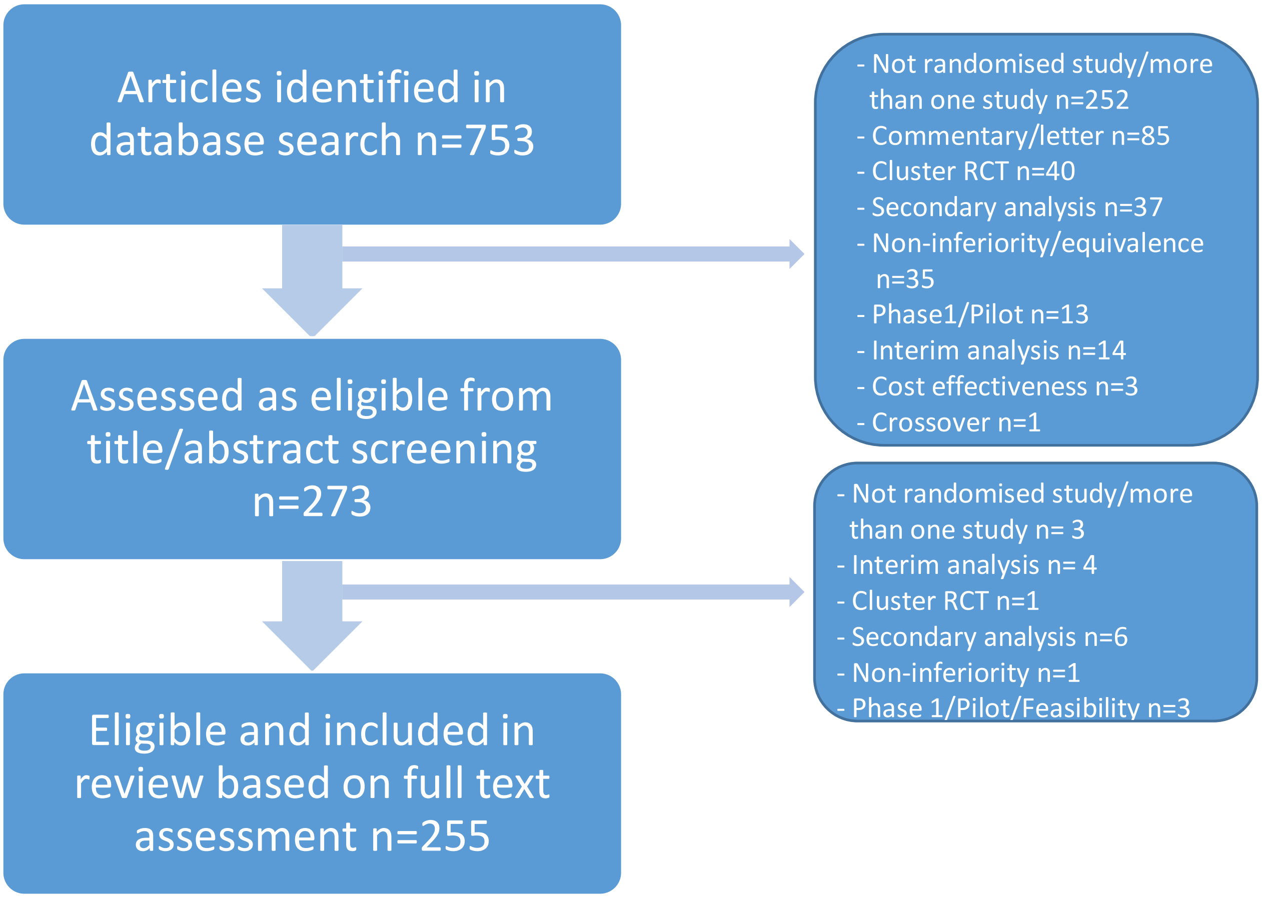

# Appendix 4: Additional Results

## eTable 1 – Characteristics of eligible trials (total number of trials 255)

| **Characteristic** | **No. of Trials** | **%** |
|---|---|---|
| Journal | | |
| Annals | 16 | 6% |
| BMJ | 12 | 5% |
| JAMA | 59 | 23% |
| Lancet | 53 | 21% |
| NEJM | 104 | 41% |
| PLOS | 11 | 4% |
| Sponsor | | |
| Academic/not-for-profit | 162 | 64% |
| Pharmaceutical/for-profit | 93 | 36% |
| Type of intervention | | |
| Multiple types | 8 | 3% |
| Other | 34 | 13% |
| Drug | 175 | 69% |
| Psychosocial, behavioural, educational | 20 | 8% |
| Surgical | 18 | 7% |
| Intervention delivery | | |
| Multiple long-term (>1 month) | 146 | 57% |
| Multiple mid-term (<=1 month) | 29 | 11% |
| Multiple short-term (≤1 week) | 15 | 6% |
| One off | 54 | 21% |
| Unclear | 11 | 4% |
| Pragmatic† trial | | |
| No | 240 | 94% |
| Yes | 15 | 6% |
| No. of treatment arms | | |
| 2 | 213 | 84% |
| 3 | 25 | 10% |
| 4 | 8 | 3% |
| 5 or more | 9 | 4% |
| Outcome type | | |
| Binary | 89 | 35% |
| Continuous | 76 | 30% |
| Count/rate | 10 | 4% |
| Ordinal | 9 | 4% |
| Survival/time-to-event | 71 | 28% |
| Covid-19 trial | | |
| No | 227 | 89% |
| Yes | 28 | 11% |
| Protocol available (in English) | | |
| No | 23 | 9% |
| Yes | 232 | 91% |
| Sample size | | |
| Median, IQR | 402 | (225, 984) |

†Trial self-defined design as pragmatic.

## eTable 2 – Reasons why population, treatment condition and population level summary measure attributes were stated, inferable or non-inferable

| Attribute and description | Trials n/N (%) |
|---|---|
| **Population** | |
| Stated by trial authors (N=4) | |
| All eligible participants[a] | 4/4 (100%) |
| Inferable (N=169) | |
| Inferred as all eligible participants based on ITT description/analysis set | 165/169 (98%) |
| Inferred as all eligible participants with a specified baseline characteristic based on ITT description for a particular cohort | 2/169 (1%) |
| Inferred as all eligible participants with a specified baseline characteristic based on analysis set description | 2/169 (1%) |
| Not inferable (N=82) | |
| Analysis population not clearly described | 7/82 (9%) |
| Post intercurrent event data not collected by trial design and analysis population not clearly described | 1/82 (1%) |
| Participants with treatment deviations excluded, but unclear whether target population was all patients (under hypothetical compliance) or subset of compliers | 56/82 (68%) |
| Participants with protocol-deviations excluded, but unclear whether target population was all patients (under hypothetical compliance) or subset of protocol compliers | 6/82 (7%) |
| Participants with other post-randomisation event excluded, but unclear whether target population was all patients (under hypothetical scenario) or subset | 12/82 (15%) |
| **Treatment condition** | |
| Stated (N=4) | |
| Specified treatment policy[b] | 1/4 (25%) |
| Specified treatment policy, given no other specified treatment[c] | 1/4 (25%) |
| Specified treatment, given full adherence with no ancillary treatment[d] | 1/4 (25%) |
| Specified treatment, given full adherence[e] | 1/4 (25%) |
| Inferable (N=223) | |
| Inferred as initial randomised treatment offer based on ITT/analysis description | 174/223 (78%) |
| Inferred as an intended treatment based on exclusion of certain deviations from analysis population | 49/223 (22%) |
| Not inferable (N=28) | |
| Unclear how treatment deviations will be handled in analysis | 22/28 (79%) |
| Unclear which treatment strategy planned analysis corresponds to[f] | 5/28 (18%) |
| Data from treatment deviators not collected by trial design and unclear how treatment deviations will be handled in analysis | 1/28 (4%) |
| **Population-level summary measure** | |
| Inferable (N=224) | |
| Inferred from type of analysis model | 77/224 (34%) |
| Stated type of summary measure they would estimate | 147/224 (66%) |
| Not inferable (N=31) | |
| Analysis strategy not clearly described | 16/31 (52%) |
| Statistical test only | 15/31 (48%) |

[a] Statements included: (i) "Target population consists of persons with type 1 diabetes that meets the inclusion/exclusion criteria", (ii) "The 'trial product' estimand evaluates the treatment effect (difference in change of A1C from baseline to week 26) between once-weekly insulin icodec and once-daily IGlar U100 for all randomised participants", (iii) "The primary objective of the trial was to assess the benefit of maintenance avelumab therapy over control therapy in prolonging overall survival among all the patients who had undergone randomisation (overall population)", and (iv) "The primary estimand of interest is called the efficacy estimand and is the effect of the randomised treatments in all subjects assuming continuation of randomised treatments for the duration of the study regardless of actual compliance".b Statement: 'a treatment policy/de-factor estimand approach was applied'. C Statement: "Non-adherence to study drug schedule" and "Permanent discontinuation of study drug" and "use of prohibited medication" were "Ignored" and "ESKD diagnosis or treatment" are " imputed under MNAR assumption". d Statement: "the trial-product estimand, defined as the between-group difference in the change in glycated hemoglobin level from baseline to week 26 among all patients who underwent randomisation, had all patients

continued to receive the trial product without receiving ancillary therapies." e Statement ""The primary estimand of interest is called the efficacy estimand and is the effect of the randomised treatments in all subjects assuming continuation of randomised treatments for the duration of the study regardless of actual compliance".[f]n=4 specified a per-protocol analysis but did not further indicate what this meant regarding treatment deviations and N=1 specified an ITT analysis with LOCF for post treatment deviation data, unclear exactly what treatment condition of interest (treatment policy or hypothetical).

## eTable 3 – Reasons why handling of intercurrent events were stated, inferable or non-inferable

| Attribute and description[§] | Trials n/N (%) |
|---|---|
| **Non-adherence – no reason (N=165)** | |
| Stated (n=5) | |
| Hypothetical | 2/5 (40%) |
| Composite | 1/5 (20%) |
| Treatment policy | 2/5 (40%) |
| Inferable (n=126) | |
| Analysis by ITT/m-ITT, inferred as treatment policy | 123/126 (97%) |
| Set as treatment failures, inferred as composite | 1/126 (1%) |
| Censored at time of non-adherence in time-to-event analysis, inferred as hypothetical | 1/126 (1%) |
| Set post IE data missing and applied multiple imputation under MAR, inferred as hypothetical | 1/126 (1%) |
| Not inferable (n=34) | |
| Unclear how treatment deviations were handled in analysis | 6/34 (18%) |
| Participants with non-adherence excluded, but unclear whether intention was to target hypothetical or principal stratum strategy | 28/34 (82%) |
| **Non-adherence – due to adverse event (N=111)** | |
| Stated (n=5) | |
| Hypothetical | 2/5 (40%) |
| Composite | 1/5 (20%) |
| Treatment policy | 2/5 (40%) |
| Inferable (n=94) | |
| Analysis by ITT/m-ITT, inferred as treatment policy | 84/94 (90%) |
| Set as treatment failures, inferred as composite | 7/94 (7%) |
| Set as poor outcome (percentage change in outcome to 0), inferred as composite | 1/94 (1%) |
| Set missing post IE and applied multiple imputation under MAR, inferred as hypothetical | 1/94 (1%) |
| Censored at time of non-adherence in time-to-event analysis, inferred as hypothetical | 1/94 (1%) |
| Not inferable (n=12) | |
| Unclear how treatment deviations were handled in analysis | 8/12 (67%) |
| Participants with non-adherence due to ae excluded, but unclear whether intention was to target hypothetical or principal stratum strategy | 4/12 (33%) |
| **Non-adherence – due to other reasons (N=133)** | |
| Stated (n=3) | |
| Hypothetical | 2/3 (67%) |
| Composite | 1/3 (33%) |
| Inferable (n=100) | |
| Analysis by ITT, inferred as treatment policy | 89/100 (89%) |
| Set as treatment failures, inferred as composite | 8/100 (8%) |
| Set missing post IE and applied multiple imputation under MAR, inferred as hypothetical | 1/100 (1%) |
| Censored at time of non-adherence in time-to-event analysis, inferred as hypothetical | 1/100 (1%) |

| | |
|---|---|
| Censored at last follow-up time in time-to-event analysis, inferred as composite (representing infinite time to event)[b] | 1/100 (1%) |
| Not inferable (n=30) | |
| Unclear how treatment deviations were handled in analysis | 11/30 (37%) |
| Participants with non-adherence due to ae excluded, but unclear whether intention was to target hypothetical or principal stratum strategy | 19/30 (63%) |
| **Additional non-trial treatment (N=29)** | |
| Stated (n=2) | |
| Treatment policy | 2/2 (100%) |
| Inferable (n=23) | |
| Analysis by ITT, inferred as treatment policy | 13/23 (57%) |
| Set as treatment failures, inferred as composite | 7/23 (30%) |
| Set as poor outcome (percentage change in outcome to 0), inferred as composite | 1/23 (4%) |
| Set post IE data missing and applied mixed model for repeated measures under MAR, inferred as hypothetical | 1/23 (4%) |
| Censored at time of treatment commencement in time-to-event analysis, inferred as hypothetical | 1/23 (4%) |
| Not inferable (n=4) | |
| Unclear how non-trial treatment use were handled in analysis | 2/4 (50%) |
| Participants with non-trial treatment use excluded, but unclear whether intention was to target hypothetical or principal stratum strategy | 2/4 (50%) |
| **Treatment switching (N=39)** | |
| Stated (n=1) | |
| Composite | 1/1 (100%) |
| Inferable (n=35) | |
| Analysis by ITT, inferred as treatment policy | 32/35 (91%) |
| Set as treatment failures, inferred as composite | 2/35 (6%) |
| Censored at last follow-up time in time-to-event analysis, inferred as composite (representing infinite time to event)[b] | 1/35 (3%) |
| Not inferable (n=3) | |
| Unclear how treatment switching was handled in analysis | 1/3 (33%) |
| Participants who switched treatment excluded, but unclear whether intention was to target hypothetical or principal stratum strategy | 2/3 (67%) |
| **Mortality (N=97)** | |
| Stated (n=1) | |
| Hypothetical | 1/1 (100%) |
| Inferable (n=23) | |
| Censored at time of death in time-to-event analysis, inferred as hypothetical | 14/23 (61%) |
| Joint model for continuous outcome and time to death, inferred as hypothetical[a] | 1/23 (4%) |
| Censored at last follow-up time in time-to-event analysis, inferred as of composite (representing infinite time to event)[b] | 3/23 (13%) |
| Competing-risk marginal models for re-current events, inferred as hypothetical[c] | 2/23 (9%) |
| Used placebo based multiple imputation post-death, inferred as hypothetical | 1/23 (4%) |
| Assigned worst score of 0 [continuous score], inferred as composite | 1/23 (4%) |
| Assigned failure, inferred as composite | 1/23 (4%) |
| Not inferable (n=73) | |
| Unclear how mortality was handled in analysis | 68/73 (93%) |
| Participants who had died excluded, but unclear whether intention was to target hypothetical or principal stratum strategy | 5/73 (7%) |
| **Other terminal event (N=5)** | |

| | |
|---|---|
| Not inferable (n=5) | |
| Participants who had event excluded, but unclear whether intention was to target hypothetical or principal stratum strategy | 5/5 (100%) |
| **Other intercurrent event 1 (N=44)** | |
| Stated (n=3) | |
| Hypothetical | 2/3 (67%) |
| Treatment policy | 1/3 (33%) |
| Not stated but inferable (n=26) | |
| Analysis by ITT, inferred as treatment policy | 21/26 (81%) |
| Censored at time of event in time-to-event analysis, inferred as hypothetical | 2/26 (8%) |
| Joint model for continuous outcome and time to event, inferred as hypothetical[a] | 1/26 (4%) |
| Set as treatment failures, inferred as composite | 2/26 (8%) |
| Not inferable (n=15) | |
| Unclear how event was handled in analysis | 1/15 (7%) |
| Participants who had event excluded, but unclear whether intention was to target hypothetical or principal stratum strategy | 14/15 (93%) |
| **Other intercurrent event 2 (N=11)** | |
| Not stated but inferable (n=6) | |
| Analysis by ITT, inferred as treatment policy | 6/6 (100%) |
| Not inferable (n=5) | |
| Unclear how event was handled in analysis | 1/5 (20%) |
| Participants who had event excluded, but unclear whether intention was to target hypothetical or principal stratum strategy | 4/5 (80%) |
| **Other intercurrent event 3 (N=2)** | |
| Not stated but inferable (n=1) | |
| Analysis by ITT, inferred as treatment policy | 1/1 (100%) |
| Not inferable (n=1) | |
| Participants who had event excluded, but unclear whether intention was to target hypothetical or principal stratum strategy | 1/1 (100%) |
| **Handling of all intercurrent events that occurred (N=242)** | |
| All relevant IEs stated | 4/242 (2%) |
| All relevant IEs not stated, but inferable | 121/242 (50%) |
| One or more relevant IE not stated or inferable | 117/242 (48%) |

[§]Statements by trial authors are presented in Table S4. [a,b,c] were considered difficult to infer, see Table S4 for more details on these methods.

## eTable 4 – Listing of other intercurrent events by trial (44 Trials)

| Other intercurrent event(s) |
|---|
| Antiparkinson medication (allowed by protocol/part of usual care) |
| Use of subsequent Iron chelation therapy (ICT) after study treatment discontinuation |
| End-stage kidney disease |
| Covid symptomatic on day 1 |
| ESKD diagnosis or treatment (hemodialysis or transplant) |
| (1) Thyroid hormones, (2) sulfur-containing imidazole derivatives for the treatment of Graves' disease |
| Dose interruption due to adverse event |
| Changed the mode of CETA delivery from group to individual counseling |
| TAVI (surgery) did not happen |
| Recommendation to receive study Trt regardless of randomly assigned group following first interim analysis |
| Dose modifications due to AE |
| Ancillary treatment |
| (1) Wound reported or identified during course of the trial (excluding wounds resulting from cellulitis) (2) Tinea or toe web intertrigo reported or identified during course of the trial |
| (1) Covid<14 days after second dose (early case) (2) Positive test result for covid at day 1 |
| Testing positive for HIV |
| New anticancer therapy started |
| Concomitant use of riluzole, edaravone, or both |
| (1) Kidney not transplantable (2) Kidney without suitable donor (3) Partner kidney excluded |
| Incorrect dosing (time of dose, or amount) |
| PCI (surgery) did not occur |
| (1) Postponed surgery (2) Unplanned indication for ICU or postoperative ventilation |
| Non-issue of bottle of best-dose vitamin D to those in other non-control groups |
| receiving supplemental oxygen through a nasal cannula during blood gas analysis |
| (1) Cancelled surgery (2) Surgery occurred outside of prescribed timelines |
| Potential unmasking |
| Emergency pleural procedure |
| (1) Non-viable of pregnancy (2) Primary infection ruled out (falsely interpreted cytomegalovirus serology) |
| Negative covid test result |
| Cannula changed out of protocol for anatomical reasons |
| Received treatment <5 days |
| (1) Dose modification due to adverse event (2) Incidence of onset of chronic GVHD (competing risk) |
| Failure to escalate to specified dose |
| Covid within 7 days of second dose |
| (1) Use of glucocorticoids (2) Use of hydroxuchloroquine (3) Unblinding |
| Covid-19 occurrence in country [COVID pandemic] |
| Surgery withheld |
| Surgery cancelled |
| Operated off-pump (surgical trial) |
| Adverse event [leading to exclusion from analysis] |
| Did not meet inclusion criteria based on central pathology review |
| (1) Open label remdesivir, azithromycin and corticosteroids (allowable medication) (2) Laboratory confirmed Covid |
| (1) Confirmed Covid (2) Dose modification |
| Early recurrence and repeat procedure required |
| Dose reduction |

## eTable 5 - The handling of intercurrent events by type of event

| Intercurrent event | N trials | % |
|---|---|---|
| **Non-adherence - no reason (N=131)** | | |
| Composite | 1 | 1% |
| Hypothetical | 4 | 3% |
| Treatment policy | 126 | 96% |
| **Non-adherence - due to adverse event (N=99)** | | |
| Composite | 9 | 9% |
| Hypothetical | 4 | 4% |
| Treatment policy | 86 | 87% |
| **Non-adherence - due to other reasons (N=103)** | | |
| Composite | 9 | 9% |
| Hypothetical | 4 | 4% |
| Treatment policy | 90 | 87% |
| **Additional non-trial treatment (N=25)** | | |
| Composite | 6 | 24% |
| Hypothetical | 1 | 4% |
| Treatment policy | 18 | 72% |
| **Treatment switching (N=36)** | | |
| Composite | 4 | 11% |
| Treatment policy | 32 | 89% |
| **Mortality (N=24)** | | |
| Composite | 5 | 21% |
| Hypothetical | 19 | 79% |
| **Other intercurrent event 1 (N=29)** | | |
| Composite | 2 | 7% |
| Hypothetical | 5 | 17% |
| Treatment policy | 22 | 76% |
| **Other intercurrent event 2 (N=6)** | | |
| Treatment policy | 6 | 100% |
| **Other intercurrent event 3 (N=1)** | | |
| Treatment policy | 1 | 100% |

## eTable 6 - Statements provided by trial authors for handling intercurrent events

| Handling of intercurrent event statements |
|---|
| **Non-adherence – no reason (N=5)** |
| "The primary estimand of interest was the efficacy estimand, which assumed continuation of randomised treatments for the trial duration, regardless of actual compliance." |
| "the trial-product estimand, defined as the between-group difference in the change in glycated hemoglobin level from baseline to week 26 among all patients who underwent randomisation, had all patients continued to receive the trial product without receiving ancillary therpies." |
| "The composite strategy, which was applied to categorical efficacy and health outcome variables, indicated that any intercurrent events—eg, discontinuing treatment or switching to open-label treatment—were to be assigned unfavourable values (ie, non-responder). Thus, patients were considered non-responders at timepoints when they did not meet the clinical response criteria or when they had missing clinical response data." |
| "a treatment policy/de-facto estimand approach was applied for the primary analysis that included all observed data irrespective of subject adherence to the randomised treatment.<br>Patients who withdrew from their randomised treatment before week 24 were followed up " |
| [Group A event includes Non-adherence to study drug schedule/permanent discontinuation of study drug]<br>"Group A IC events were considered as directly interpretable. Effectively, IC events in this group are ignored, in agreement with the intention-to-treat (ITT) principle" |
| **Non-adherence – due to adverse event (N=5)** |
| "The primary estimand of interest was the efficacy estimand, which assumed continuation of randomised treatments for the trial duration, regardless of actual compliance." |
| "the trial-product estimand, defined as the between-group difference in the change in glycated hemoglobin level from baseline to week 26 among all patients who underwent randomisation, had all patients continued to receive the trial product without receiving ancillary therpies." |
| "The composite strategy, which was applied to categorical efficacy and health outcome variables, indicated that any intercurrent events—eg, discontinuing treatment or switching to open-label treatment—were to be assigned unfavourable values (ie, non-responder). Thus, patients were considered non-responders at timepoints when they did not meet the clinical response criteria or when they had missing clinical response data." |
| "a treatment policy/de-facto estimand approach was applied for the primary analysis that included all observed data irrespective of subject adherence to the randomised treatment.<br>Patients who withdrew from their randomised treatment before week 24 were followed up " |
| [Group A event includes Non-adherence to study drug schedule/permanent discontinuation of study drug]<br>"Group A IC events were considered as directly interpretable. Effectively, IC events in this group are ignored, in agreement with the intention-to-treat (ITT) principle" |
| **Non-adherence – due to other reasons (N=3)** |
| "The primary estimand of interest was the efficacy estimand, which assumed continuation of randomised treatments for the trial duration, regardless of actual compliance." |
| "the trial-product estimand, defined as the between-group difference in the change in glycated hemoglobin level from baseline to week 26 among all patients who underwent randomisation, had all patients continued to receive the trial product without receiving ancillary therapies." |
| "The composite strategy, which was applied to categorical efficacy and health outcome variables, indicated that any intercurrent events—eg, discontinuing treatment or switching to open-label treatment—were to be assigned unfavourable values (ie, non-responder). Thus, patients were considered non-responders at timepoints when they did not meet the clinical response criteria or when they had missing clinical response data." |
| **Additional non-trial treatment (N=2)** |
| "A treatment policy/de-facto estimand approach was applied for the primary analysis that included all observed data irrespective of subject adherence to the randomised treatment. Patients who withdrew from their randomised treatment before week 24 were followed up through to week 24, and their week 24 data were considered as primary, irrespective of treatment discontinuation, treatment interruptions, or use of rescue medication (WLL or other)." |
| [Group A event includes use of prohibited medication ] |

| |
|---|
| “Group A IC events were considered as directly interpretable. Effectively, IC events in this group are ignored, in agreement with the intention-to-treat (ITT) principle” |
| **Treatment switching (N=1)** |
| “The composite strategy, which was applied to categorical efficacy and health outcome variables, indicated that any intercurrent events—eg, discontinuing treatment or switching to open-label treatment—were to be assigned unfavourable values (ie, non-responder). Thus, patients were considered non-responders at timepoints when they did not meet the clinical response criteria or when they had missing clinical response data.” “Patients who discontinued the masked study treatment to which they were originally assigned and switched to open-label ixekizumab Q2W were considered non-responders after switching.” |
| **Mortality (N=1)** |
| [Group C event = Terminal event, i.e., death]<br>“Group C IC events were assumed to conform to a *hypothetical* scenario, in which post-IC iGFR values have a similar distribution to other non-ESKD subjects with similar characteristics and pre-IC iGFR values.” |
| **Other intercurrent event 1 (N=3)** |
| [Group B event = ESKD diagnosis or treatment ]<br>“Group B IC events were assumed to follow a *hypothetical* scenario, in which iGFR values after developing ESKD take on biologically plausible values that are not confounded by the IC event, i.e., by ESKD treatments such as dialysis or kidney transplant. “ |
| [Other IE = anciallary treatment] “The ‘trial product’ estimand evaluates the treatment effect (difference in change of A1C from baseline to week 26) between once-weekly insulin icodec and once-daily IGlar U100 for all randomised participants, under the assumption that all participants had adhered to treatment for the entire planned duration of the trial and did not receive ancillary treatment. This is a ‘hypothetical’ estimand intended to provide an estimation of the achievable treatment effect of insulin icodec without any confounding effect of ancillary treatment for participants that are actually able to take the drug during the intended treatment period.” |
| [Other IE = supplemental oxygen through a nasal cannula during blood gas analysis] “a treatment policy/de-facto estimand approach was applied for the primary analysis that included all observed data irrespective of subject adherence to the randomised treatment. Patients who withdrew from their randomised treatment before week 24 were followed up through to week 24, and their week 24 data were considered as primary, irrespective of treatment discontinuation, treatment interruptions, or use of rescue medication (WLL or other).” |

## eTable 7 - Subgroup analysis by sponsor (academic/not for profit versus pharmaceutical/for profit)

| **Outcome** | **Academic/not for profit** | | **Pharmaceutical/for profit** | |
|---|---|---|---|---|
| | **N** | **%** | **N** | **%** |
| Any potential/actual IEs described | | | | |
| No | 6 | 4% | 0 | 0% |
| Yes | 156 | 96% | 93 | 100% |
| Any IE frequency data reported | | | | |
| No | 7 | 4% | 0 | 0% |
| Yes | 155 | 96% | 93 | 100% |
| IE frequency ≥1 | | | | |
| No | 11 | 7% | 2 | 2% |
| Yes | 151 | 93% | 91 | 98% |
| ICH E9 R1 addendum referenced | | | | |
| No | 160 | 99% | 90 | 97% |
| Yes | 2 | 1% | 3 | 3% |
| Estimand term used | | | | |
| No | 161 | 99% | 90 | 97% |
| Yes | 1 | 1% | 3 | 3% |
| Overall estimand | | | | |
| Estimand inferable | 83 | 51% | 34 | 37% |
| Estimand not inferable | 79 | 49% | 59 | 63% |
| No. of attributes not stated or inferable | | | | |
| 0 | 83 | 51% | 34 | 37% |
| 1 | 33 | 20% | 21 | 23% |
| 2 | 23 | 14% | 27 | 29% |
| 3 | 21 | 13% | 10 | 11% |
| 4 | 2 | 1% | 1 | 1% |
| Population stated | | | | |
| Neither stated nor inferable | 46 | 28% | 36 | 39% |
| Not stated, but inferable | 115 | 71% | 54 | 58% |
| Stated | 1 | 1% | 3 | 3% |
| Treatment condition(s) stated | | | | |
| Neither stated nor inferable | 19 | 12% | 9 | 10% |
| Not stated, but inferable | 142 | 88% | 81 | 87% |
| Stated | 1 | 1% | 3 | 3% |
| Outcome stated | | | | |
| Stated | 162 | 100% | 93 | 100% |
| Handling of intercurrent events stated | | | | |
| Neither stated nor inferable | 68 | 42% | 49 | 53% |
| Not stated, but inferable | 82 | 51% | 39 | 42% |
| Stated | 1 | 1% | 3 | 3% |
| Unclear if any intercurrent events occurred/relevant | 11 | 7% | 2 | 2% |
| Population-level summary measure stated | | | | |
| Neither stated nor inferable | 17 | 10% | 14 | 15% |
| Not stated, but inferable | 145 | 90% | 79 | 85% |
| If stated or inferable; Population | N=116 | | N=57 | |
| All eligible participants | 115 | 99% | 54 | 95% |
| All eligible participants with a pre-specified baseline characteristic | 1 | 1% | 3 | 5% |
| If stated or inferable; Treatment condition(s) | N=143 | | N=84 | |
| Initiating treatment, with no rescue medication | 0 | 0% | 1 | 1% |
| Treatment offer regardless of any IEs (treatment policy) | 123 | 86% | 52 | 62% |

| | | | | |
|---|---|---|---|---|
| Initiating treatment | 13 | 9% | 23 | 27% |
| Receiving all treatment | 3 | 2% | 5 | 6% |
| Receiving a specific amount of treatment | 0 | 0% | 1 | 1% |
| Receiving all treatment, with no other specified treatment | 0 | 0% | 2 | 2% |
| Treatment offer, given received a specified surgery/procedure | 3 | 2% | 0 | 0% |
| Treatment offer, with no other specified treatment | 1 | 1% | 0 | 0% |
| Outcome type | | | | |
| Binary | 60 | 37% | 29 | 31% |
| Continuous | 51 | 31% | 25 | 27% |
| Count/rate | 4 | 2% | 6 | 6% |
| Ordinal | 7 | 4% | 2 | 2% |
| Survival | 40 | 25% | 31 | 33% |
| Composite outcome incorporating/potentially incorporating an IE | | | | |
| No | 124 | 77% | 74 | 80% |
| Yes | 38 | 23% | 19 | 20% |
| If stated or inferable; strategy for handling intercurrent events exclusive treatment policy | N=83 | | N=42 | |
| No | 15 | 18% | 14 | 33% |
| Yes | 68 | 82% | 28 | 67% |
| If stated or inferable; Strategy for handling intercurrent events | | | | |
| Composite | 1 | 1% | 4 | 10% |
| Hypothetical | 1 | 1% | 1 | 2% |
| Treatment policy | 68 | 82% | 28 | 67% |
| Treatment policy & composite | 5 | 6% | 2 | 5% |
| Treatment policy & hypothetical | 8 | 10% | 7 | 17% |
| If unclear whether intercurrent events; Infer strategy for handling | N=11 | | N=2 | |
| No (i.e. if not ITT) | 2 | 18% | 0 | 0% |
| Yes (i.e. if ITT) | 9 | 82% | 2 | 100% |
| If stated or inferable; Population level summary | N=145 | | N=79 | |
| 1-HR | 0 | 0% | 1 | 1% |
| 1-IRR | 0 | 0% | 1 | 1% |
| 1-RR | 0 | 0% | 1 | 1% |
| HR | 37 | 26% | 29 | 37% |
| IRR | 3 | 2% | 0 | 0% |
| OR | 9 | 6% | 8 | 10% |
| OR - proportional odds | 6 | 4% | 2 | 3% |
| RD | 21 | 14% | 5 | 6% |
| RR | 20 | 14% | 4 | 5% |
| SMD | 2 | 1% | 0 | 0% |
| geometric mean ratio | 1 | 1% | 0 | 0% |
| mean difference | 40 | 28% | 23 | 29% |
| mean difference AUC | 1 | 1% | 0 | 0% |
| median difference | 5 | 3% | 0 | 0% |
| median ratio | 0 | 0% | 1 | 1% |
| rate ratio | 0 | 0% | 4 | 5% |
| Any supplementary estimands | N=162 | | N=93 | |
| No | 80 | 49% | 63 | 68% |
| Yes | 82 | 51% | 30 | 32% |
| Number of supplementary estimands | N=82 | | N=30 | |
| 1 | 57 | 70% | 23 | 77% |
| 2 | 20 | 24% | 5 | 17% |
| 3 | 4 | 5% | 0 | 0% |
| 4 | 1 | 1% | 2 | 7% |
| One or more supplementary estimand stated/inferable | | | | |
| No | 63 | 77% | 21 | 70% |

| | | | | |
|---|---|---|---|---|
| Yes | 19 | 23% | 9 | 30% |
| No. supplementary estimand stated/inferable | | | | |
| 0 | 63 | 77% | 21 | 70% |
| 1 | 18 | 22% | 8 | 27% |
| 2 | 0 | 0% | 1 | 3% |
| 4 | 1 | 1% | 0 | 0% |
| One or more IE strategy stated/inferable | | | | |
| No | 59 | 72% | 19 | 63% |
| Yes | 23 | 28% | 11 | 37% |
| No. IE strategy stated/inferable supplementary estimand | | | | |
| 0 | 59 | 72% | 19 | 63% |
| 1 | 21 | 26% | 8 | 27% |
| 2 | 1 | 1% | 2 | 7% |
| 3 | 0 | 0% | 1 | 3% |
| 4 | 1 | 1% | 0 | 0% |
| Treatment policy exclusive IE supplementary estimand stated/inferable | | | | |
| No | 78 | 95% | 27 | 90% |
| Yes | 4 | 5% | 3 | 10% |
| One or more other IE supplementary estimands | | | | |
| No | 63 | 77% | 21 | 70% |
| Yes | 19 | 23% | 9 | 30% |
| No. of other (non-treatment policy) supplementary estimands | | | | |
| 0 | 63 | 77% | 21 | 70% |
| 1 | 17 | 21% | 7 | 23% |
| 2 | 1 | 1% | 1 | 3% |
| 3 | 0 | 0% | 1 | 3% |
| 4 | 1 | 1% | 0 | 0% |

Treatment policy refers to planned treatment course – not necessarily received.

## eTable 8 - Subgroup analysis by pragmatic trial design

| Outcome | Not pragmatic trial | | Pragmatic trial | |
|---|---|---|---|---|
| | N | % | N | % |
| Any potential/actual IEs described | N=240 | | N=15 | |
| No | 5 | 2% | 1 | 7% |
| Yes | 235 | 98% | 14 | 93% |
| Any IE frequency data reported | | | | |
| No | 6 | 3% | 1 | 7% |
| Yes | 234 | 98% | 14 | 93% |
| IE frequency ≥1 | | | | |
| No | 10 | 4% | 3 | 20% |
| Yes | 230 | 96% | 12 | 80% |
| ICH E9 R1 addendum referenced | | | | |
| No | 235 | 98% | 15 | 100% |
| Yes | 5 | 2% | 0 | 0% |
| Estimand term used | | | | |
| No | 236 | 98% | 15 | 100% |
| Yes | 4 | 2% | 0 | 0% |
| Overall estimand | | | | |
| Estimand inferable | 109 | 45% | 8 | 53% |
| Estimand not inferable | 131 | 55% | 7 | 47% |
| No. of attributes not stated or inferable | | | | |
| 0 | 109 | 45% | 8 | 53% |
| 1 | 51 | 21% | 3 | 20% |
| 2 | 49 | 20% | 1 | 7% |
| 3 | 28 | 12% | 3 | 20% |
| 4 | 3 | 1% | 0 | 0% |
| Population stated | | | | |
| Neither stated nor inferable | 78 | 33% | 4 | 27% |
| Not stated, but inferable | 158 | 66% | 11 | 73% |
| Stated | 4 | 2% | 0 | 0% |
| Treatment condition(s) stated | | | | |
| Neither stated nor inferable | 25 | 10% | 3 | 20% |
| Not stated, but inferable | 211 | 88% | 12 | 80% |
| Stated | 4 | 2% | 0 | 0% |
| Outcome stated | | | | |
| Stated | 240 | 100% | 15 | 100% |
| Handling of intercurrent events stated | | | | |
| Neither stated nor inferable | 110 | 46% | 7 | 47% |
| Not stated, but inferable | 116 | 48% | 5 | 33% |
| Stated | 4 | 2% | 0 | 0% |
| Unclear if any intercurrent events occurred/relevant | 10 | 4% | 3 | 20% |
| Population-level summary measure stated | | | | |
| Neither stated nor inferable | 31 | 13% | 0 | 0% |
| Not stated, but inferable | 209 | 87% | 15 | 100% |
| If stated or inferable; Population | N=162 | | N=11 | |
| All eligible participants | 159 | 98% | 10 | 91% |
| All eligible participants with a pre-specified baseline characteristic | 3 | 2% | 1 | 9% |
| If stated or inferable; Treatment condition(s) | N=215 | | N=12 | |
| Initiating treatment, with no rescue medication | 1 | 0% | 0 | 0% |
| Treatment offer regardless of any IEs (treatment policy) | 164 | 76% | 11 | 92% |
| Initiating treatment | 35 | 16% | 1 | 8% |
| Receiving all treatment | 8 | 4% | 0 | 0% |

| | | | | |
|---|---|---|---|---|
| Receiving a specific amount of treatment | 1 | 0% | 0 | 0% |
| Receiving all treatment, with no other specified treatment | 2 | 1% | 0 | 0% |
| Treatment offer, given received a specified surgery/procedure | 3 | 1% | 0 | 0% |
| Treatment offer, with no other specified treatment | 1 | 0% | 0 | 0% |
| Outcome type | | | | |
| Binary | 83 | 35% | 6 | 40% |
| Continuous | 71 | 30% | 5 | 33% |
| Count/rate | 9 | 4% | 1 | 7% |
| Ordinal | 8 | 3% | 1 | 7% |
| Survival | 69 | 29% | 2 | 13% |
| Composite outcome incorporating/potentially incorporating an IE | | | | |
| No | 185 | 77% | 13 | 87% |
| Yes | 55 | 23% | 2 | 13% |
| If stated or inferable; Strategy for handling intercurrent events exclusively treatment policy | N=120 | | N=5 | |
| No | 29 | 24% | 0 | 0% |
| Yes | 91 | 76% | 5 | 100% |
| If stated or inferable; Strategy for handling intercurrent events | | | | |
| Composite | 5 | 4% | 0 | 0% |
| Hypothetical | 2 | 2% | 0 | 0% |
| Treatment policy | 91 | 75% | 5 | 100% |
| Treatment policy & composite | 7 | 6% | 0 | 0% |
| Treatment policy & hypothetical | 15 | 12% | 0 | 0% |
| If unclear whether intercurrent events; Infer strategy for handling | N=10 | | N=3 | |
| No (i.e. if not ITT) | 2 | 20% | 0 | 0% |
| Yes (i.e. if ITT) | 8 | 80% | 3 | 100% |
| If stated or inferable; Population level summary | N=209 | | N=15 | |
| 1-HR | 1 | 0% | 0 | 0% |
| 1-IRR | 1 | 0% | 0 | 0% |
| 1-RR | 1 | 0% | 0 | 0% |
| HR | 64 | 31% | 2 | 13% |
| IRR | 2 | 1% | 1 | 7% |
| OR | 17 | 8% | 0 | 0% |
| OR - proportional odds | 7 | 3% | 1 | 7% |
| RD | 24 | 11% | 2 | 13% |
| RR | 20 | 10% | 4 | 27% |
| SMD | 2 | 1% | 0 | 0% |
| geometric mean ratio | 1 | 0% | 0 | 0% |
| mean difference | 58 | 28% | 5 | 33% |
| mean difference AUC | 1 | 0% | 0 | 0% |
| median difference | 5 | 2% | 0 | 0% |
| median ratio | 1 | 0% | 0 | 0% |
| rate ratio | 4 | 2% | 0 | 0% |
| Any supplementary estimands | N=240 | | N=15 | |
| No | 137 | 57% | 6 | 40% |
| Yes | 103 | 43% | 9 | 60% |
| Number | N=103 | | N=9 | |
| 1 | 73 | 71% | 7 | 78% |
| 2 | 23 | 22% | 2 | 22% |
| 3 | 4 | 4% | 0 | 0% |
| 4 | 3 | 3% | 0 | 0% |

| | | | | |
|---|---|---|---|---|
| One or more supplementary estimand stated/inferable | | | | |
| No | 78 | 76% | 6 | 67% |
| Yes | 25 | 24% | 3 | 33% |
| No. supplementary estimand stated/inferable | | | | |
| 0 | 78 | 76% | 6 | 67% |
| 1 | 23 | 22% | 3 | 33% |
| 2 | 1 | 1% | 0 | 0% |
| 4 | 1 | 1% | 0 | 0% |
| One or more IE strategy stated/inferable | | | | |
| No | 74 | 72% | 4 | 44% |
| Yes | 29 | 28% | 5 | 56% |
| No. IE strategy stated/inferable supplementary estimand | | | | |
| 0 | 74 | 72% | 4 | 44% |
| 1 | 24 | 23% | 5 | 56% |
| 2 | 3 | 3% | 0 | 0% |
| 3 | 1 | 1% | 0 | 0% |
| 4 | 1 | 1% | 0 | 0% |
| One or more treatment policy exclusive IE supplementary estimand stated/inferable | | | | |
| No | 96 | 93% | 9 | 100% |
| Yes | 7 | 7% | 0 | 0% |
| No. treatment policy exclusive supplementary estimands stated/inferable | | | | |
| 0 | 96 | 93% | 9 | 100% |
| 1 | 7 | 7% | 0 | 0% |
| One or more other IE supplementary estimands | | | | |
| No | 80 | 78% | 4 | 44% |
| Yes | 23 | 22% | 5 | 56% |
| No. of other (non-treatment policy) supplementary estimands | | | | |
| 0 | 80 | 78% | 4 | 44% |
| 1 | 19 | 18% | 5 | 56% |
| 2 | 2 | 2% | 0 | 0% |
| 3 | 1 | 1% | 0 | 0% |
| 4 | 1 | 1% | 0 | 0% |

Treatment policy refers to planned treatment course – not necessarily received.

## eTable 9 – Analysis where inferring of the strategy for handling intercurrent events was difficult

| Analysis | Details to inform inferability |
|---|---|
| Primary estimand | |
| One trial specified analysis followed the ITT principle and used a joint model for a continuous outcome (change in the estimated glomerular filtration rate (eGFR) from baseline modelled using linear mixed model) and time to trial discontinuation due to death or end stage kidney disease before end of 104 week follow-up (Weibull parametric survival model). | We reached consensus that this inferred a hypothetical estimand strategy with respect to death/end stage kidney since the continuous outcome is not collected post the occurrence of death/end stage kidney disease (terminal IEs where the measurement no longer exists) and the resulting treatment effect is estimated conditional on a patient-specific frailty (random effect) that models the correlation between the continuous outcome and occurrence of death/end stage kidney disease. Following ITT principle inferred treatment policy with respect to non-terminal intercurrent events. |
| Four trials used an outcome of time to recovery or clinical improvement over a pre-define follow-up period (28 or 29 days) and for patients who did not recover and died prior to the end of the follow-up period right-censored the data at the last follow-up day. Analysis followed ITT principle. | This is equivalent to setting death to an infinite recovery/improvement time, as clarified in a reference given by one of the three trials "We note that, with time-to-improvement/recovery models, the competing event of death requires special handling. Patients who die during follow-up should not be censored at time of death, as that assumes their recovery time would be like all who remain alive and unrecovered at that time. To state the obvious, once dead, a patient cannot recover. A death must be set to an infinite recovery time, so that at the end of follow-up, the patient is counted as "not recovered. We achieve the same objective by censoring deaths at the last observation day. Therefore, patients censored on the last observation day reflect two different states: death and failure to recover by day 28" [2]." This handles the competing event of death in a similar manner to the Fine and Gray competing risk approach (see Table 4). Therefore we reached consensus that this approach and the Fine and Gray approach (Table 4) inferred a composite strategy with respect to death. Following ITT principle, inferred treatment policy with respect to non-terminal intercurrent events. |
| Two trials specified analysis followed the ITT principle and used Competing-risk marginal models for re-current events to handle terminal competing events | We reached consensus that this inferred a hypothetical strategy with respect to the handling of the specified terminal intercurrent events, since the recurrent event can no longer occur post the occurrence of the terminal competing events and the resulting treatment effect is estimated conditional on a patient-specific frailty that models the correlation between the count outcome and occurrence of the competing event. This concurs with Krol et al. who explored the use of the joint frailty model with recurrent event when targeting the hypothetical estimand [3]. Following ITT principle inferred treatment policy with respect to non-terminal intercurrent events. |
| Supplementary estimand | |

| | |
|---|---|
| Fine and Gray Model | We reached consensus that this approach inferred a composite strategy with respect to the handling of deaths as described by [2]. When implemented with an ITT analysis strategy we inferred treatment policy with respect to the non-terminal intercurrent events. |

## eTable 10 - Supplementary IE estimand details including strategies and analysis (n=154 supplementary estimands in 112 trials)

| **Supplementary estimand outcome** | **No. of Trials** | **%** |
|---|---|---|
| Estimand term used | | |
| No | 152 | 99% |
| Yes | 2 | 1% |
| Overall estimand | | |
| Estimand inferable | 32 | 21% |
| Estimand not inferable | 122 | 79% |
| No. of attributes not stated or inferable | | |
| 0 | 32 | 21% |
| 1 | 23 | 15% |
| 2 | 78 | 51% |
| 3 | 17 | 11% |
| 4 | 4 | 3% |
| Population stated | | |
| Neither stated nor inferable | 97 | 63% |
| Not stated, but inferable | 56 | 36% |
| Stated | 1 | 1% |
| Treatment condition(s) stated | | |
| Neither stated nor inferable | 30 | 20% |
| Not stated, but inferable | 122 | 79% |
| Stated | 2 | 1% |
| Outcome stated | | |
| Stated | 154 | 100% |
| Handling of intercurrent events stated | | |
| Neither stated nor inferable | 111 | 73% |
| Not stated, but inferable | 41 | 27% |
| Stated | 1 | 1% |
| Population-level summary measure stated | | |
| Neither stated nor inferable | 12 | 8% |
| Not stated but inferable | 142 | 92% |
| If stated or inferable; Population | N=57 | |
| Principal stratum of defined treatment compliers | 7 | 12% |
| All eligible participants | 49 | 86% |
| All eligible participants with a pre-specified baseline characteristic | 1 | 2% |
| If stated or inferable; Treatment condition(s) | N=124 | |
| Receiving all treatment | 30 | 24% |
| Treatment offer regardless of any IEs (treatment policy) | 29 | 23% |
| Receiving a specific amount of treatment | 24 | 21% |
| Initiating treatment | 17 | 13% |
| Treatment offer, with no other specified treatment | 9 | 7% |
| Receiving all treatment, with no other specified treatment | 4 | 4% |
| Receiving a specific amount of treatment, with no other specified treatment | 3 | 3% |
| Treatment offer, with no switching | 2 | 2% |
| Initiating treatment, with no specified treatment | 1 | 1% |
| Initiating treatment, with no switching | 1 | 1% |
| Treatment offer, given received individual counselling | 1 | 1% |
| Receiving treatment, with no switching | 1 | 1% |

| | | |
|---|---|---|
| Receiving all treatment & surgery/procedure | 1 | 1% |
| The change in treatment effect per unit change in compliance | 1 | 1% |
| Composite outcome incorporating/potentially incorporating an IE | | |
| No | 121 | 79% |
| Yes (death) | 33 | 21% |
| If stated or inferable; Strategy for handling intercurrent events Trt policy | N=42 | |
| No | 35 | 83% |
| Yes | 7 | 17% |
| If stated or inferable; Strategy for handling intercurrent events | | |
| Hypothetical | 3 | 7% |
| Principal stratum | 7 | 17% |
| Treatment policy | 7 | 17% |
| Treatment policy & composite | 11 | 26% |
| Treatment policy & hypothetical | 7 | 17% |
| Treatment policy & hypothetical & composite | 4 | 10% |
| Treatment policy + hypothetical | 3 | 7% |
| If stated or inferable; Population level summary | N=142 | |
| 1-HR | 1 | 1% |
| 1-RR | 1 | 1% |
| HR | 31 | 22% |
| OR | 12 | 8% |
| OR - proportional odds | 9 | 6% |
| RD | 10 | 7% |
| RR | 17 | 12% |
| Subdistributional HR | 9 | 6% |
| mean difference | 50 | 35% |
| rate ratio | 2 | 1% |

## eTable 11 - Summary of estimand use in Protocols or SAPs

| Protocol/SAP outcome | No. of Trials | % |
|---|---|---|
| Protocol or SAP available* in English | N=255 | |
| no | 24 | 9% |
| yes | 231 | 91% |
| If Protocol/SAP available, Estimand term used in Protocol/SAP | N=231 | |
| no | 213 | 92% |
| yes | 18 | 8% |
| If Protocol/SAP available, Protocol/SAP referenced ICH E9 R1 | | |
| No | 228 | 99% |
| Yes | 3 | 1% |
| If Protocol/SAP available, Primary Estimand use in Protocol/SAP | | |
| Fully stated | 4 | 2% |
| Included 'estimand' but not defined | 4§ | 2% |
| Not mentioned/defined | 213 | 92% |
| Partially stated | 10 | 4% |
| If Protocol/SAP available, Supplementary Estimand use in Protocol/SAP | | |
| Fully stated | 0 | 0% |
| Mentioned but not defined | 1 | 0% |
| Not mentioned/defined | 224 | 97% |
| Partially stated | 5 | 2% |
| Referenced ICH-E9 R1 only | 1 | 0% |
| Protocol available | N=255 | |
| No | 24 | 9% |
| Yes | 231 | 91% |
| If Protocol available, Estimand term used Protocol | N=231 | |
| No | 220 | 95% |
| Yes | 11 | 5% |
| If Protocol available, Protocol referenced ICH E9 R1 | | |
| No | 230 | 100% |
| Yes | 1 | 0% |
| If Protocol available, Primary Estimand use in Protocol | | |
| Fully stated estimand | 3 | 1% |
| Mentioned but not defined | 2 | 1% |
| Not mentioned/defined | 220 | 95% |
| Partially stated estimand | 6 | 3% |
| If Protocol available, Supplementary Estimand use in Protocol | | |
| Fully stated estimand | 5 | 2% |
| Mentioned but not defined | 2 | 1% |
| Not mentioned/defined | 220 | 95% |
| Partially stated estimand | 4 | 2% |
| SAP available | | |
| No | 105 | 41% |
| Yes | 150 | 59% |
| If SAP available, Estimand term used SAP | N=150 | |

| | | |
|---|---|---|
| No | 134 | 89% |
| Yes | 16 | 11% |
| If SAP available, SAP referenced ICH E9 R1 | | |
| No | 148 | 99% |
| Yes | 2 | 1% |
| If SAP available, Primary Estimand use in SAP | | |
| Fully stated | 3 | 2% |
| Mentioned but not defined | 2 | 1% |
| Not mentioned/defined | 134 | 89% |
| Partially stated | 10 | 7% |
| Referenced ICH-E9 only | 1 | 1% |
| If SAP available, Supplementary Estimand use in SAP | | |
| Fully stated | 0 | 0% |
| Mentioned but not defined | 1 | 1% |
| Not mentioned/defined | 144 | 95% |
| Partially stated | 4 | 3% |
| Referenced ICH-E9 only | 1 | 1% |

*As supplementary material or published/available on a website and referenced in the article.

§n=1 Referenced ICH-E9 R1 only; n=1 said would use “ITT estimand” (primary)/”on-treatment estimand” (supplement) but did not define either of these estimands further; n=1 said “a de-facto estimand approach” will be applied but did not define this estimand; n=1 said would use an “effectiveness estimand” but did not define this estimand.

## eTable 12 - Primary Estimand stated/partially stated in Protocol or SAP (n=14)

| Primary Estimand definitions | No. of Trials | % |
|---|---|---|
| Estimand definition stated in Protocol/SAP | | |
| Fully stated | 4 | 29% |
| Partially stated | 10 | 71% |
| Where stated/partially stated | | |
| Both Protocol & SAP | 8† | 57% |
| Protocol only | 1 | 7% |
| Sap only | 5 | 36% |
| No. attributes not stated | | |
| 0 | 4 | 29% |
| 1 | 3 | 21% |
| 2 | 7 | 50% |
| Population stated | | |
| No | 9* | 64% |
| Yes | 5 | 36% |
| Treatment stated | | |
| No | 2 | 14% |
| Yes | 12 | 86% |
| Outcome stated | | |
| Yes | 14 | 100% |
| Any IE handling stated | | |
| Yes | 14 | 100% |
| If IE handling stated (n=14), did IE handling stated cover all realized IE's that occurred? | | |
| No | 8 | 57% |
| Yes | 6 | 43% |
| Population level summary stated | | |
| No | 6[¥] | 43% |
| Yes | 8 | 57% |

Estimand attributes identical across SAPS and protocols where relevant. IE = Intercurrent event.*Eight trials attempted a population definition but we classified this as not stated as this referred to the analysis population as follows: a) Subjects who are randomised and received at least 1 dose of investigational product (FAS), b) All randomised subjects, c) All randomised subjects (FAS), d) Full analysis set, e) All randomised subjects who received at least one dose (FAS) f) Modified Intent-to-Treat population g) All randomised subjects who received at least one dose of double-blinded BMN 111 or placebo (Protocol) v FAS (defined as all randomised subjects in the SAP) h) In all subjects. [¥]One trial specified the population-level summary measure attribute as "Population-average treatment effect on eGFR at 4 months after randomization." But we classified this as not stated as information on the estimator was required to infer the actual population level summary measure.

## eTable 13 Estimand attributes stated by the trial authors in protocol/SAP populations:

| **Population stated by trialists** |
|---|
| Individuals with T1D meeting the inclusion/exclusion criteria specified in the Study Protocol. |
| Adults aged 18 years and older in circumstances at a high risk of SARS-CoV-2 infection but without medical conditions that pose additional risk of developing severe disease. [& list of other exclusions.] |
| Population of Patients with heart failure with reduced ejection fraction |
| Healthy adults after 1 or 2 doses |
| Population: Defined through appropriate inclusion/exclusion criteria (see Section 6.1 and 6.2) to reflect the targeted patient population |
| **Treatment conditions stated by trialists** |
| Treatment effect due to the initially randomised treatments as actually taken |
| The initially assigned and dosed investigational product (anifrolumab and placebo) |
| Initiating treatment with semaglutide as compared to placebo |
| If had adhered to treatment and did not receive ancillary (1/wk v 1/daily Iglar) |
| Regardless of treatment adherence (evolocumab vs placebo) |
| The effect of the initially assigned randomised study drug, |
| Test: mRNA-1273. Reference: Placebo [given] receiving the second dose of IP per protocol schedule |
| Empagliflozin 10 mg and placebo regardless of changes of treatment (including discontinuation of trial medication) until completion of the planned treatment phase |
| Regardless of adherence to treatment and subsequent therapies |
| Randomised treatments……assuming continuation of randomised treatments for the duration of the study regardless of actual compliance |
| Complying with receipt of second dose |
| Measurement of intervention effect: Regardless of stopping study treatment or adherence to study treatment. |
| **Intercurrent events handling stated by trialists** |
| Group A will be considered as directly interpretable. Effectively, IC events in this group are ignored, which is consistent with the ITT principle.", Group B are assumed to follow a hypothetical scenario, in which variable of interest after developing ESRD takes on biologically plausible values that are not confounded by IC event i.e. by ESRD treatment Group C are assumed to conform to a hypothetical scenario in which post-IC values of the variable of interest (or endpoint) have a similar distribution to other non-ESRD subjects [Group A events = Non-adherence to study drug schedule, Permanent discontinuation of study drug, use of prohibited medication, missed scheduled visit. Group B = ESRD treatment, Group C = Early discontinuation from the study, Terminal event i.e. death] |
| Discontinuation of investigational product and receipt of restricted medications are unfavourable outcomes |
| De-facto (effectiveness) treatment effect |
| Hypothetical (if all had adhered and did not initiate ancillary) |
| Regardless of treatment adherence |
| Irrespective of adherence |
| Composite [Treatment failure, discontinuation, withdrawal, lost, death] |
| Unrelated death - While alive/ Early infection - Treatment policy/ Missed dose of IP - Principal stratum/ SARS-CoV-2 – Principal stratum. |
| Regardless of changes of treatment (including discontinuation of trial medication) |
| Regardless of adherence to treatment and subsequent therapies. |

| |
|---|
| Regardless of whether or not switching to rescue medication had occurred or subjects had discontinued from the study. |
| Assuming continuation of randomised treatments |
| Complying with receipt of second dose; hypothetical setting where participants follow the study schedules and protocol requirements as directed |
| Regardless of stopping study treatment or adherence to study treatment. |

| **Stated population level summary** | **No. of Trials** |
|---|---|
| Difference in proportions | 1 |
| OR | 1 |
| 1-HR | 1 |
| HR | 1 |
| Difference in means | 3 |
| 1-IRR | 1 |

## eTable 14 - Primary Estimands stated/partially stated in Protocol (n=9)

| **Protocol primary estimand definition** | **No. of Trials** | **%** |
|---|---|---|
| Estimand stated in Protocol | | |
| Fully stated | 3 | 33% |
| Partially stated | 6 | 67% |
| No. attributes not stated | | |
| 0 | 3 | 33% |
| 1 | 2 | 22% |
| 2 | 3 | 33% |
| 3 | 1 | 11% |
| Population stated | | |
| No | 6* | 67% |
| Yes | 3 | 33% |
| Treatment stated | | |
| No | 1 | 11% |
| Yes | 8 | 89% |
| Outcome stated | | |
| Yes | 9 | 100% |
| Any IE handling stated | | |
| No | 1 | 11% |
| Yes | 8 | 89% |
| Population level summary stated | | |
| No | 3 | 33% |
| Yes | 6 | 67% |

*Six trials attempted a population definition but we classified this as not stated as this referred to the analysis population

## eTable 15 - Primary estimands stated/partially stated in SAP (n=13)

| **SAP primary estimand definitions** | **No. of Trials** | **%** |
|---|---|---|
| Estimand stated in SAP | | |
| Fully stated | 3 | 23% |
| Partially stated | 10 | 77% |
| No. attributes not stated | | |
| 0 | 3 | 23% |
| 1 | 3 | 23% |
| 2 | 7 | 54% |
| Population stated | | |
| No | 9* | 69% |
| Yes | 4 | 31% |
| Treatment stated | | |
| No | 2 | 15% |
| Yes | 11 | 85% |
| Outcome stated | | |
| Yes | 13 | 100% |
| Any IE handling stated | | |
| Yes | 13 | 100% |
| Population level summary stated | | |
| No | 6[¥] | 46% |
| Yes | 7 | 54% |

*Eight trials attempted a population definition but we classified this as not stated as this referred to the analysis population. [¥]One trial specified the population-level summary measure attribute as "Population-average treatment effect on eGFR at 4 months after randomization." But we classified this as not stated as information on the estimator was required to infer the actual population level summary measure.

## Appendix 6 – List of reviewed articles (n=255)

1. HALT-IT Trial Collaborators, Effects of a high-dose 24-h infusion of tranexamic acid on death and thromboembolic events in patients with acute gastrointestinal bleeding (HALT-IT): an international randomised, double-blind, placebo-controlled trial. Lancet (London, England). 2020;395(10241):1927-36.
2. Reinforcement of Closure of Stoma Site (ROCSS) Collaborative and West Midlands Research Collaborative, Prophylactic biological mesh reinforcement versus standard closure of stoma site (ROCSS): a multicentre, randomised controlled trial. Lancet (London, England). 2020;395(10222):417-26.
3. The HIP ATTACK Investigators, Accelerated surgery versus standard care in hip fracture (HIP ATTACK): an international, randomised, controlled trial. Lancet (London, England). 2020;395(10225):698-708.
4. The Parkinson Study Group STEADY-PD III Investigators, Isradipine Versus Placebo in Early Parkinson Disease: A Randomized Trial. Annals of internal medicine. 2020;172(9):591-8.
5. Agarwal A, Mukherjee A, Kumar G, Chatterjee P, Bhatnagar T, Malhotra P. Convalescent plasma in the management of moderate covid-19 in adults in India: open label phase II multicentre randomised controlled trial (PLACID Trial). BMJ (Clinical research ed). 2020;371:m3939.
6. Aldoss I, La Rosa C, Baden LR, Longmate J, Ariza-Heredia EJ, Rida WN, et al. Poxvirus Vectored Cytomegalovirus Vaccine to Prevent Cytomegalovirus Viremia in Transplant Recipients: A Phase 2, Randomized Clinical Trial. Annals of internal medicine. 2020;172(5):306-16.
7. Ali MK, Chwastiak L, Poongothai S, Emmert-Fees KMF, Patel SA, Anjana RM, et al. Effect of a Collaborative Care Model on Depressive Symptoms and Glycated Hemoglobin, Blood Pressure, and Serum Cholesterol Among Patients With Depression and Diabetes in India: The INDEPENDENT Randomized Clinical Trial. Jama. 2020;324(7):651-62.
8. Allen KD, Woolson S, Hoenig HM, Bongiorni D, Byrd J, Caves K, et al. Stepped Exercise Program for Patients With Knee Osteoarthritis : A Randomized Controlled Trial. Annals of internal medicine. 2020.
9. Alsan M, Stanford FC, Banerjee A, Breza E, Chandrasekhar AG, Eichmeyer S, et al. Comparison of Knowledge and Information-Seeking Behavior After General COVID-19 Public Health Messages and Messages Tailored for Black and Latinx Communities : A Randomized Controlled Trial. Annals of internal medicine. 2020.
10. Amarenco P, Kim JS, Labreuche J, Charles H, Abtan J, Béjot Y, et al. A Comparison of Two LDL Cholesterol Targets after Ischemic Stroke. The New England journal of medicine. 2020;382(1):9.
11. Amstutz A, Nsakala BL, Vanobberghen F, Muhairwe J, Glass TR, Namane T, et al. Switch to second-line versus continued first-line antiretroviral therapy for patients with low-level HIV-1 viremia: An open-label randomized controlled trial in Lesotho. PLoS medicine. 2020;17(9):e1003325.
12. Andrade JG, Wells GA, Deyell MW, Bennett M, Essebag V, Champagne J, et al. Cryoablation or Drug Therapy for Initial Treatment of Atrial Fibrillation. The New England journal of medicine. 2021;384(4):305-15.
13. Angelucci E, Li J, Greenberg P, Wu D, Hou M, Montano Figueroa EH, et al. Iron Chelation in Transfusion-Dependent Patients With Low- to Intermediate-1-Risk Myelodysplastic Syndromes: A Randomized Trial. Annals of internal medicine. 2020;172(8):513-22.
14. Antoszyk AN, Glassman AR, Beaulieu WT, Jampol LM, Jhaveri CD, Punjabi OS, et al. Effect of Intravitreous Aflibercept vs Vitrectomy With Panretinal Photocoagulation on Visual Acuity in Patients With Vitreous Hemorrhage From Proliferative Diabetic Retinopathy: A Randomized Clinical Trial. Jama. 2020;324(23):2383-95.
15. Appel LJ, Michos ED, Mitchell CM, Blackford AL, Sternberg AL, Miller Iii ER, et al. The Effects of Four Doses of Vitamin D Supplements on Falls in Older Adults : A Response-Adaptive, Randomized Clinical Trial. Annals of internal medicine. 2020.

16. Arabi YM, Asiri AY, Assiri AM, Balkhy HH, Al Bshabshe A, Al Jeraisy M, et al. Interferon Beta-1b and Lopinavir-Ritonavir for Middle East Respiratory Syndrome. The New England journal of medicine. 2020;383(17):1645-56.
17. Armstrong PW, Lam CSP, Anstrom KJ, Ezekowitz J, Hernandez AF, O'Connor CM, et al. Effect of Vericiguat vs Placebo on Quality of Life in Patients With Heart Failure and Preserved Ejection Fraction: The VITALITY-HFpEF Randomized Clinical Trial. Jama. 2020;324(15):1512-21.
18. Armstrong PW, Pieske B, Anstrom KJ, Ezekowitz J, Hernandez AF, Butler J, et al. Vericiguat in Patients with Heart Failure and Reduced Ejection Fraction. The New England journal of medicine. 2020;382(20):1883-93.
19. Baden LR, El Sahly HM, Essink B, Kotloff K, Frey S, Novak R, et al. Efficacy and Safety of the mRNA-1273 SARS-CoV-2 Vaccine. The New England journal of medicine. 2021;384(5):403-16.
20. Badve SV, Pascoe EM, Tiku A, Boudville N, Brown FG, Cass A, et al. Effects of Allopurinol on the Progression of Chronic Kidney Disease. The New England journal of medicine. 2020;382(26):2504-13.
21. Bagshaw SM, Wald R, Adhikari NKJ, Bellomo R, da Costa BR, Dreyfuss D, et al. Timing of Initiation of Renal-Replacement Therapy in Acute Kidney Injury. The New England journal of medicine. 2020;383(3):240-51.
22. Bailey CS, Rasoulinejad P, Taylor D, Sequeira K, Miller T, Watson J, et al. Surgery versus Conservative Care for Persistent Sciatica Lasting 4 to 12 Months. The New England journal of medicine. 2020;382(12):1093-102.
23. Bakris GL, Agarwal R, Anker SD, Pitt B, Ruilope LM, Rossing P, et al. Effect of Finerenone on Chronic Kidney Disease Outcomes in Type 2 Diabetes. The New England journal of medicine. 2020;383(23):2219-29.
24. Balwani M, Sardh E, Ventura P, Peiró PA, Rees DC, Stölzel U, et al. Phase 3 Trial of RNAi Therapeutic Givosiran for Acute Intermittent Porphyria. The New England journal of medicine. 2020;382(24):2289-301.
25. Bangalore S, Maron DJ, O'Brien SM, Fleg JL, Kretov EI, Briguori C, et al. Management of Coronary Disease in Patients with Advanced Kidney Disease. The New England journal of medicine. 2020;382(17):1608-18.
26. Barrot L, Asfar P, Mauny F, Winiszewski H, Montini F, Badie J, et al. Liberal or Conservative Oxygen Therapy for Acute Respiratory Distress Syndrome. The New England journal of medicine. 2020;382(11):999-1008.
27. Baxter BT, Matsumura J, Curci JA, McBride R, Larson L, Blackwelder W, et al. Effect of Doxycycline on Aneurysm Growth Among Patients With Small Infrarenal Abdominal Aortic Aneurysms: A Randomized Clinical Trial. Jama. 2020;323(20):2029-38.
28. Beigel JH, Tomashek KM, Dodd LE, Mehta AK, Zingman BS, Kalil AC, et al. Remdesivir for the Treatment of Covid-19 - Final Report. The New England journal of medicine. 2020;383(19):1813-26.
29. Bhatnagar R, Piotrowska HEG, Laskawiec-Szkonter M, Kahan BC, Luengo-Fernandez R, Pepperell JCT, et al. Effect of Thoracoscopic Talc Poudrage vs Talc Slurry via Chest Tube on Pleurodesis Failure Rate Among Patients With Malignant Pleural Effusions: A Randomized Clinical Trial. Jama. 2020;323(1):60-9.
30. Bhatt DL, Szarek M, Pitt B, Cannon CP, Leiter LA, McGuire DK, et al. Sotagliflozin in Patients with Diabetes and Chronic Kidney Disease. The New England journal of medicine. 2021;384(2):129-39.
31. Bhatt DL, Szarek M, Steg PG, Cannon CP, Leiter LA, McGuire DK, et al. Sotagliflozin in Patients with Diabetes and Recent Worsening Heart Failure. The New England journal of medicine. 2021;384(2):117-28.
32. Birtle A, Johnson M, Chester J, Jones R, Dolling D, Bryan RT, et al. Adjuvant chemotherapy in upper tract urothelial carcinoma (the POUT trial): a phase 3, open-label, randomised controlled trial. Lancet (London, England). 2020;395(10232):1268-77.

33. Bischoff-Ferrari HA, Vellas B, Rizzoli R, Kressig RW, da Silva JAP, Blauth M, et al. Effect of Vitamin D Supplementation, Omega-3 Fatty Acid Supplementation, or a Strength-Training Exercise Program on Clinical Outcomes in Older Adults: The DO-HEALTH Randomized Clinical Trial. Jama. 2020;324(18):1855-68.
34. Blanc FX, Badje AD, Bonnet M, Gabillard D, Messou E, Muzoora C, et al. Systematic or Test-Guided Treatment for Tuberculosis in HIV-Infected Adults. The New England journal of medicine. 2020;382(25):2397-410.
35. Bonaca MP, Bauersachs RM, Anand SS, Debus ES, Nehler MR, Patel MR, et al. Rivaroxaban in Peripheral Artery Disease after Revascularization. The New England journal of medicine. 2020;382(21):1994-2004.
36. Boulware DR, Pullen MF, Bangdiwala AS, Pastick KA, Lofgren SM, Okafor EC, et al. A Randomized Trial of Hydroxychloroquine as Postexposure Prophylaxis for Covid-19. The New England journal of medicine. 2020;383(6):517-25.
37. Breton MD, Kanapka LG, Beck RW, Ekhlaspour L, Forlenza GP, Cengiz E, et al. A Randomized Trial of Closed-Loop Control in Children with Type 1 Diabetes. The New England journal of medicine. 2020;383(9):836-45.
38. Bundgaard H, Bundgaard JS, Raaschou-Pedersen DET, von Buchwald C, Todsen T, Norsk JB, et al. Effectiveness of Adding a Mask Recommendation to Other Public Health Measures to Prevent SARS-CoV-2 Infection in Danish Mask Wearers : A Randomized Controlled Trial. Annals of internal medicine. 2020.
39. Burke CA, Dekker E, Lynch P, Samadder NJ, Balaguer F, Hüneburg R, et al. Eflornithine plus Sulindac for Prevention of Progression in Familial Adenomatous Polyposis. The New England journal of medicine. 2020;383(11):1028-39.
40. Burmester GR, Buttgereit F, Bernasconi C, Álvaro-Gracia JM, Castro N, Dougados M, et al. Continuing versus tapering glucocorticoids after achievement of low disease activity or remission in rheumatoid arthritis (SEMIRA): a double-blind, multicentre, randomised controlled trial. Lancet (London, England). 2020;396(10246):267-76.
41. Butler CC, Lau M, Gillespie D, Owen-Jones E, Lown M, Wootton M, et al. Effect of Probiotic Use on Antibiotic Administration Among Care Home Residents: A Randomized Clinical Trial. Jama. 2020;324(1):47-56.
42. Butler CC, van der Velden AW, Bongard E, Saville BR, Holmes J, Coenen S, et al. Oseltamivir plus usual care versus usual care for influenza-like illness in primary care: an open-label, pragmatic, randomised controlled trial. Lancet (London, England). 2020;395(10217):42-52.
43. Cai G, Aitken D, Laslett LL, Pelletier JP, Martel-Pelletier J, Hill C, et al. Effect of Intravenous Zoledronic Acid on Tibiofemoral Cartilage Volume Among Patients With Knee Osteoarthritis With Bone Marrow Lesions: A Randomized Clinical Trial. Jama. 2020;323(15):1456-66.
44. Campbell BCV, Mitchell PJ, Churilov L, Yassi N, Kleinig TJ, Dowling RJ, et al. Effect of Intravenous Tenecteplase Dose on Cerebral Reperfusion Before Thrombectomy in Patients With Large Vessel Occlusion Ischemic Stroke: The EXTEND-IA TNK Part 2 Randomized Clinical Trial. Jama. 2020;323(13):1257-65.
45. Cao B, Wang Y, Wen D, Liu W, Wang J, Fan G, et al. A Trial of Lopinavir-Ritonavir in Adults Hospitalized with Severe Covid-19. The New England journal of medicine. 2020;382(19):1787-99.
46. Cappellini MD, Viprakasit V, Taher AT, Georgiev P, Kuo KHM, Coates T, et al. A Phase 3 Trial of Luspatercept in Patients with Transfusion-Dependent β-Thalassemia. The New England journal of medicine. 2020;382(13):1219-31.
47. Cavalcanti AB, Zampieri FG, Rosa RG, Azevedo LCP, Veiga VC, Avezum A, et al. Hydroxychloroquine with or without Azithromycin in Mild-to-Moderate Covid-19. The New England journal of medicine. 2020;383(21):2041-52.
48. Chalmers JD, Haworth CS, Metersky ML, Loebinger MR, Blasi F, Sibila O, et al. Phase 2 Trial of the DPP-1 Inhibitor Brensocatib in Bronchiectasis. The New England journal of medicine. 2020;383(22):2127-37.

49. Chalmers JR, Haines RH, Bradshaw LE, Montgomery AA, Thomas KS, Brown SJ, et al. Daily emollient during infancy for prevention of eczema: the BEEP randomised controlled trial. Lancet (London, England). 2020;395(10228):962-72.
50. Chamberlain JM, Kapur J, Shinnar S, Elm J, Holsti M, Babcock L, et al. Efficacy of levetiracetam, fosphenytoin, and valproate for established status epilepticus by age group (ESETT): a double-blind, responsive-adaptive, randomised controlled trial. Lancet (London, England). 2020;395(10231):1217-24.
51. Charles P, Perrodeau É, Samson M, Bonnotte B, Néel A, Agard C, et al. Long-Term Rituximab Use to Maintain Remission of Antineutrophil Cytoplasmic Antibody-Associated Vasculitis: A Randomized Trial. Annals of internal medicine. 2020;173(3):179-87.
52. Chen GL, Coates EE, Plummer SH, Carter CA, Berkowitz N, Conan-Cibotti M, et al. Effect of a Chikungunya Virus-Like Particle Vaccine on Safety and Tolerability Outcomes: A Randomized Clinical Trial. Jama. 2020;323(14):1369-77.
53. Choi IJ, Kim CG, Lee JY, Kim YI, Kook MC, Park B, et al. Family History of Gastric Cancer and Helicobacter pylori Treatment. The New England journal of medicine. 2020;382(5):427-36.
54. Chu JJ, Devall AJ, Beeson LE, Hardy P, Cheed V, Sun Y, et al. Mifepristone and misoprostol versus misoprostol alone for the management of missed miscarriage (MifeMiso): a randomised, double-blind, placebo-controlled trial. Lancet (London, England). 2020;396(10253):770-8.
55. Cohen CR, Wierzbicki MR, French AL, Morris S, Newmann S, Reno H, et al. Randomized Trial of Lactin-V to Prevent Recurrence of Bacterial Vaginosis. The New England journal of medicine. 2020;382(20):1906-15.
56. Conaghan PG, Bowes MA, Kingsbury SR, Brett A, Guillard G, Rizoska B, et al. Disease-Modifying Effects of a Novel Cathepsin K Inhibitor in Osteoarthritis: A Randomized Controlled Trial. Annals of internal medicine. 2020;172(2):86-95.
57. Costa ML, Achten J, Knight R, Bruce J, Dutton SJ, Madan J, et al. Effect of Incisional Negative Pressure Wound Therapy vs Standard Wound Dressing on Deep Surgical Site Infection After Surgery for Lower Limb Fractures Associated With Major Trauma: The WHIST Randomized Clinical Trial. Jama. 2020;323(6):519-26.
58. Costa ML, Achten J, Marian IR, Dutton SJ, Lamb SE, Ollivere B, et al. Plaster cast versus functional brace for non-surgical treatment of Achilles tendon rupture (UKSTAR): a multicentre randomised controlled trial and economic evaluation. Lancet (London, England). 2020;395(10222):441-8.
59. Croop R, Lipton RB, Kudrow D, Stock DA, Kamen L, Conway CM, et al. Oral rimegepant for preventive treatment of migraine: a phase 2/3, randomised, double-blind, placebo-controlled trial. Lancet (London, England). 2021;397(10268):51-60.
60. Dangas GD, Tijssen JGP, Wöhrle J, Søndergaard L, Gilard M, Möllmann H, et al. A Controlled Trial of Rivaroxaban after Transcatheter Aortic-Valve Replacement. The New England journal of medicine. 2020;382(2):120-9.
61. de Bono J, Mateo J, Fizazi K, Saad F, Shore N, Sandhu S, et al. Olaparib for Metastatic Castration-Resistant Prostate Cancer. The New England journal of medicine. 2020;382(22):2091-102.
62. de Koning HJ, van der Aalst CM, de Jong PA, Scholten ET, Nackaerts K, Heuvelmans MA, et al. Reduced Lung-Cancer Mortality with Volume CT Screening in a Randomized Trial. The New England journal of medicine. 2020;382(6):503-13.
63. Dellon ES, Peterson KA, Murray JA, Falk GW, Gonsalves N, Chehade M, et al. Anti-Siglec-8 Antibody for Eosinophilic Gastritis and Duodenitis. The New England journal of medicine. 2020;383(17):1624-34.
64. Deodhar A, Helliwell PS, Boehncke WH, Kollmeier AP, Hsia EC, Subramanian RA, et al. Guselkumab in patients with active psoriatic arthritis who were biologic-naive or had previously received TNFα inhibitor treatment (DISCOVER-1): a double-blind, randomised, placebo-controlled phase 3 trial. Lancet (London, England). 2020;395(10230):1115-25.

65. Deodhar A, van der Heijde D, Gensler LS, Kim TH, Maksymowych WP, Østergaard M, et al. Ixekizumab for patients with non-radiographic axial spondyloarthritis (COAST-X): a randomised, placebo-controlled trial. Lancet (London, England). 2020;395(10217):53-64.
66. Dequin PF, Heming N, Meziani F, Plantefève G, Voiriot G, Badié J, et al. Effect of Hydrocortisone on 21-Day Mortality or Respiratory Support Among Critically Ill Patients With COVID-19: A Randomized Clinical Trial. Jama. 2020;324(13):1298-306.
67. Deyle GD, Allen CS, Allison SC, Gill NW, Hando BR, Petersen EJ, et al. Physical Therapy versus Glucocorticoid Injection for Osteoarthritis of the Knee. The New England journal of medicine. 2020;382(15):1420-9.
68. Dias JJ, Brealey SD, Fairhurst C, Amirfeyz R, Bhowal B, Blewitt N, et al. Surgery versus cast immobilisation for adults with a bicortical fracture of the scaphoid waist (SWIFFT): a pragmatic, multicentre, open-label, randomised superiority trial. Lancet (London, England). 2020;396(10248):390-401.
69. Dimopoulos M, Quach H, Mateos MV, Landgren O, Leleu X, Siegel D, et al. Carfilzomib, dexamethasone, and daratumumab versus carfilzomib and dexamethasone for patients with relapsed or refractory multiple myeloma (CANDOR): results from a randomised, multicentre, open-label, phase 3 study. Lancet (London, England). 2020;396(10245):186-97.
70. DiNardo CD, Jonas BA, Pullarkat V, Thirman MJ, Garcia JS, Wei AH, et al. Azacitidine and Venetoclax in Previously Untreated Acute Myeloid Leukemia. The New England journal of medicine. 2020;383(7):617-29.
71. Doria A, Galecki AT, Spino C, Pop-Busui R, Cherney DZ, Lingvay I, et al. Serum Urate Lowering with Allopurinol and Kidney Function in Type 1 Diabetes. The New England journal of medicine. 2020;382(26):2493-503.
72. Douglas RS, Kahaly GJ, Patel A, Sile S, Thompson EHZ, Perdok R, et al. Teprotumumab for the Treatment of Active Thyroid Eye Disease. The New England journal of medicine. 2020;382(4):341-52.
73. Eisenberg MJ, Hébert-Losier A, Windle SB, Greenspoon T, Brandys T, Fülöp T, et al. Effect of e-Cigarettes Plus Counseling vs Counseling Alone on Smoking Cessation: A Randomized Clinical Trial. Jama. 2020;324(18):1844-54.
74. Fenaux P, Platzbecker U, Mufti GJ, Garcia-Manero G, Buckstein R, Santini V, et al. Luspatercept in Patients with Lower-Risk Myelodysplastic Syndromes. The New England journal of medicine. 2020;382(2):140-51.
75. Ferrari R, Ford I, Fox K, Challeton JP, Correges A, Tendera M, et al. Efficacy and safety of trimetazidine after percutaneous coronary intervention (ATPCI): a randomised, double-blind, placebo-controlled trial. Lancet (London, England). 2020;396(10254):830-8.
76. Fidler S, Stöhr W, Pace M, Dorrell L, Lever A, Pett S, et al. Antiretroviral therapy alone versus antiretroviral therapy with a kick and kill approach, on measures of the HIV reservoir in participants with recent HIV infection (the RIVER trial): a phase 2, randomised trial. Lancet (London, England). 2020;395(10227):888-98.
77. Finkelstein A, Zhou A, Taubman S, Doyle J. Health Care Hotspotting - A Randomized, Controlled Trial. The New England journal of medicine. 2020;382(2):152-62.
78. Finn RS, Qin S, Ikeda M, Galle PR, Ducreux M, Kim TY, et al. Atezolizumab plus Bevacizumab in Unresectable Hepatocellular Carcinoma. The New England journal of medicine. 2020;382(20):1894-905.
79. Fishbane S, Jamal A, Munera C, Wen W, Menzaghi F. A Phase 3 Trial of Difelikefalin in Hemodialysis Patients with Pruritus. The New England journal of medicine. 2020;382(3):222-32.
80. Forno E, Bacharier LB, Phipatanakul W, Guilbert TW, Cabana MD, Ross K, et al. Effect of Vitamin D3 Supplementation on Severe Asthma Exacerbations in Children With Asthma and Low Vitamin D Levels: The VDKA Randomized Clinical Trial. Jama. 2020;324(8):752-60.
81. Franz AR, Engel C, Bassler D, Rüdiger M, Thome UH, Maier RF, et al. Effects of Liberal vs Restrictive Transfusion Thresholds on Survival and Neurocognitive Outcomes in Extremely Low-Birth-Weight Infants: The ETTNO Randomized Clinical Trial. Jama. 2020;324(6):560-70.

82. Fritz JM, Lane E, McFadden M, Brennan G, Magel JS, Thackeray A, et al. Physical Therapy Referral From Primary Care for Acute Back Pain With Sciatica : A Randomized Controlled Trial. Annals of internal medicine. 2021;174(1):8-17.
83. Fujii T, Luethi N, Young PJ, Frei DR, Eastwood GM, French CJ, et al. Effect of Vitamin C, Hydrocortisone, and Thiamine vs Hydrocortisone Alone on Time Alive and Free of Vasopressor Support Among Patients With Septic Shock: The VITAMINS Randomized Clinical Trial. Jama. 2020;323(5):423-31.
84. Furie R, Rovin BH, Houssiau F, Malvar A, Teng YKO, Contreras G, et al. Two-Year, Randomized, Controlled Trial of Belimumab in Lupus Nephritis. The New England journal of medicine. 2020;383(12):1117-28.
85. Furtado RHM, Berwanger O, Fonseca HA, Corrêa TD, Ferraz LR, Lapa MG, et al. Azithromycin in addition to standard of care versus standard of care alone in the treatment of patients admitted to the hospital with severe COVID-19 in Brazil (COALITION II): a randomised clinical trial. Lancet (London, England). 2020;396(10256):959-67.
86. Futier E, Garot M, Godet T, Biais M, Verzilli D, Ouattara A, et al. Effect of Hydroxyethyl Starch vs Saline for Volume Replacement Therapy on Death or Postoperative Complications Among High-Risk Patients Undergoing Major Abdominal Surgery: The FLASH Randomized Clinical Trial. Jama. 2020;323(3):225-36.
87. Galsky MD, Arija JÁ A, Bamias A, Davis ID, De Santis M, Kikuchi E, et al. Atezolizumab with or without chemotherapy in metastatic urothelial cancer (IMvigor130): a multicentre, randomised, placebo-controlled phase 3 trial. Lancet (London, England). 2020;395(10236):1547-57.
88. Ganmaa D, Uyanga B, Zhou X, Gantsetseg G, Delgerekh B, Enkhmaa D, et al. Vitamin D Supplements for Prevention of Tuberculosis Infection and Disease. The New England journal of medicine. 2020;383(4):359-68.
89. Garcia-Marcinkiewicz AG, Kovatsis PG, Hunyady AI, Olomu PN, Zhang B, Sathyamoorthy M, et al. First-attempt success rate of video laryngoscopy in small infants (VISI): a multicentre, randomised controlled trial. Lancet (London, England). 2020;396(10266):1905-13.
90. Geersing GJ, Hendriksen JMT, Zuithoff NPA, Roes KC, Oudega R, Takada T, et al. Effect of tailoring anticoagulant treatment duration by applying a recurrence risk prediction model in patients with venous thromboembolism compared to usual care: A randomized controlled trial. PLoS medicine. 2020;17(6):e1003142.
91. Goldman JD, Lye DCB, Hui DS, Marks KM, Bruno R, Montejano R, et al. Remdesivir for 5 or 10 Days in Patients with Severe Covid-19. The New England journal of medicine. 2020.
92. Graham DY, Canaan Y, Maher J, Wiener G, Hulten KG, Kalfus IN. Rifabutin-Based Triple Therapy (RHB-105) for Helicobacter pylori Eradication: A Double-Blind, Randomized, Controlled Trial. Annals of internal medicine. 2020;172(12):795-802.
93. Griffin MP, Yuan Y, Takas T, Domachowske JB, Madhi SA, Manzoni P, et al. Single-Dose Nirsevimab for Prevention of RSV in Preterm Infants. The New England journal of medicine. 2020;383(5):415-25.
94. Grosicki S, Simonova M, Spicka I, Pour L, Kriachok I, Gavriatopoulou M, et al. Once-per-week selinexor, bortezomib, and dexamethasone versus twice-per-week bortezomib and dexamethasone in patients with multiple myeloma (BOSTON): a randomised, open-label, phase 3 trial. Lancet (London, England). 2020;396(10262):1563-73.
95. Gutzmer R, Stroyakovskiy D, Gogas H, Robert C, Lewis K, Protsenko S, et al. Atezolizumab, vemurafenib, and cobimetinib as first-line treatment for unresectable advanced BRAF(V600) mutation-positive melanoma (IMspire150): primary analysis of the randomised, double-blind, placebo-controlled, phase 3 trial. Lancet (London, England). 2020;395(10240):1835-44.
96. Hagen S, Elders A, Stratton S, Sergenson N, Bugge C, Dean S, et al. Effectiveness of pelvic floor muscle training with and without electromyographic biofeedback for urinary incontinence in women: multicentre randomised controlled trial. BMJ (Clinical research ed). 2020;371:m3719.

97. Hallifax RJ, McKeown E, Sivakumar P, Fairbairn I, Peter C, Leitch A, et al. Ambulatory management of primary spontaneous pneumothorax: an open-label, randomised controlled trial. Lancet (London, England). 2020;396(10243):39-49.
98. Hamilton DF, Beard DJ, Barker KL, Macfarlane GJ, Tuck CE, Stoddart A, et al. Targeting rehabilitation to improve outcomes after total knee arthroplasty in patients at risk of poor outcomes: randomised controlled trial. BMJ (Clinical research ed). 2020;371:m3576.
99. Hardeman W, Mitchell J, Pears S, Van Emmenis M, Theil F, Gc VS, et al. Evaluation of a very brief pedometer-based physical activity intervention delivered in NHS Health Checks in England: The VBI randomised controlled trial. PLoS medicine. 2020;17(3):e1003046.
100. Hassett L, van den Berg M, Lindley RI, Crotty M, McCluskey A, van der Ploeg HP, et al. Digitally enabled aged care and neurological rehabilitation to enhance outcomes with Activity and MObility UsiNg Technology (AMOUNT) in Australia: A randomised controlled trial. PLoS medicine. 2020;17(2):e1003029.
101. Heerspink HJL, Stefánsson BV, Correa-Rotter R, Chertow GM, Greene T, Hou FF, et al. Dapagliflozin in Patients with Chronic Kidney Disease. The New England journal of medicine. 2020;383(15):1436-46.
102. Herbst RS, Giaccone G, de Marinis F, Reinmuth N, Vergnenegre A, Barrios CH, et al. Atezolizumab for First-Line Treatment of PD-L1-Selected Patients with NSCLC. The New England journal of medicine. 2020;383(14):1328-39.
103. Hernández Martínez G, Rodriguez ML, Vaquero MC, Ortiz R, Masclans JR, Roca O, et al. High-Flow Oxygen with Capping or Suctioning for Tracheostomy Decannulation. The New England journal of medicine. 2020;383(11):1009-17.
104. Hetland ML, Haavardsholm EA, Rudin A, Nordström D, Nurmohamed M, Gudbjornsson B, et al. Active conventional treatment and three different biological treatments in early rheumatoid arthritis: phase IV investigator initiated, randomised, observer blinded clinical trial. BMJ (Clinical research ed). 2020;371:m4328.
105. Hill MD, Goyal M, Menon BK, Nogueira RG, McTaggart RA, Demchuk AM, et al. Efficacy and safety of nerinetide for the treatment of acute ischaemic stroke (ESCAPE-NA1): a multicentre, double-blind, randomised controlled trial. Lancet (London, England). 2020;395(10227):878-87.
106. Hoffman MK, Goudar SS, Kodkany BS, Metgud M, Somannavar M, Okitawutshu J, et al. Low-dose aspirin for the prevention of preterm delivery in nulliparous women with a singleton pregnancy (ASPIRIN): a randomised, double-blind, placebo-controlled trial. Lancet (London, England). 2020;395(10220):285-93.
107. Hofman MS, Lawrentschuk N, Francis RJ, Tang C, Vela I, Thomas P, et al. Prostate-specific membrane antigen PET-CT in patients with high-risk prostate cancer before curative-intent surgery or radiotherapy (proPSMA): a prospective, randomised, multicentre study. Lancet (London, England). 2020;395(10231):1208-16.
108. Horne AW, Vincent K, Hewitt CA, Middleton LJ, Koscielniak M, Szubert W, et al. Gabapentin for chronic pelvic pain in women (GaPP2): a multicentre, randomised, double-blind, placebo-controlled trial. Lancet (London, England). 2020;396(10255):909-17.
109. Hung IF, Lung KC, Tso EY, Liu R, Chung TW, Chu MY, et al. Triple combination of interferon beta-1b, lopinavir-ritonavir, and ribavirin in the treatment of patients admitted to hospital with COVID-19: an open-label, randomised, phase 2 trial. Lancet (London, England). 2020;395(10238):1695-704.
110. Hutchinson PJ, Edlmann E, Bulters D, Zolnourian A, Holton P, Suttner N, et al. Trial of Dexamethasone for Chronic Subdural Hematoma. The New England journal of medicine. 2020;383(27):2616-27.
111. Ikematsu H, Hayden FG, Kawaguchi K, Kinoshita M, de Jong MD, Lee N, et al. Baloxavir Marboxil for Prophylaxis against Influenza in Household Contacts. The New England journal of medicine. 2020;383(4):309-20.

112. Issa Y, Kempeneers MA, Bruno MJ, Fockens P, Poley JW, Ahmed Ali U, et al. Effect of Early Surgery vs Endoscopy-First Approach on Pain in Patients With Chronic Pancreatitis: The ESCAPE Randomized Clinical Trial. Jama. 2020;323(3):237-47.
113. Ivers NM, Schwalm JD, Bouck Z, McCready T, Taljaard M, Grace SL, et al. Interventions supporting long term adherence and decreasing cardiovascular events after myocardial infarction (ISLAND): pragmatic randomised controlled trial. BMJ (Clinical research ed). 2020;369:m1731.
114. Jabbar A, Ingoe L, Junejo S, Carey P, Addison C, Thomas H, et al. Effect of Levothyroxine on Left Ventricular Ejection Fraction in Patients With Subclinical Hypothyroidism and Acute Myocardial Infarction: A Randomized Clinical Trial. Jama. 2020;324(3):249-58.
115. Jochmans I, Brat A, Davies L, Hofker HS, van de Leemkolk FEM, Leuvenink HGD, et al. Oxygenated versus standard cold perfusion preservation in kidney transplantation (COMPARE): a randomised, double-blind, paired, phase 3 trial. Lancet (London, England). 2020;396(10263):1653-62.
116. John CC, Opoka RO, Latham TS, Hume HA, Nabaggala C, Kasirye P, et al. Hydroxyurea Dose Escalation for Sickle Cell Anemia in Sub-Saharan Africa. The New England journal of medicine. 2020;382(26):2524-33.
117. Johnston SC, Amarenco P, Denison H, Evans SR, Himmelmann A, James S, et al. Ticagrelor and Aspirin or Aspirin Alone in Acute Ischemic Stroke or TIA. The New England journal of medicine. 2020;383(3):207-17.
118. Juul SE, Comstock BA, Wadhawan R, Mayock DE, Courtney SE, Robinson T, et al. A Randomized Trial of Erythropoietin for Neuroprotection in Preterm Infants. The New England journal of medicine. 2020;382(3):233-43.
119. Kabashima K, Matsumura T, Komazaki H, Kawashima M. Trial of Nemolizumab and Topical Agents for Atopic Dermatitis with Pruritus. The New England journal of medicine. 2020;383(2):141-50.
120. Kalil AC, Patterson TF, Mehta AK, Tomashek KM, Wolfe CR, Ghazaryan V, et al. Baricitinib plus Remdesivir for Hospitalized Adults with Covid-19. The New England journal of medicine. 2020.
121. Kang DH, Park SJ, Lee SA, Lee S, Kim DH, Kim HK, et al. Early Surgery or Conservative Care for Asymptomatic Aortic Stenosis. The New England journal of medicine. 2020;382(2):111-9.
122. Karalapillai D, Weinberg L, Peyton P, Ellard L, Hu R, Pearce B, et al. Effect of Intraoperative Low Tidal Volume vs Conventional Tidal Volume on Postoperative Pulmonary Complications in Patients Undergoing Major Surgery: A Randomized Clinical Trial. Jama. 2020;324(9):848-58.
123. Kelly AS, Auerbach P, Barrientos-Perez M, Gies I, Hale PM, Marcus C, et al. A Randomized, Controlled Trial of Liraglutide for Adolescents with Obesity. The New England journal of medicine. 2020;382(22):2117-28.
124. Kim BK, Hong SJ, Cho YH, Yun KH, Kim YH, Suh Y, et al. Effect of Ticagrelor Monotherapy vs Ticagrelor With Aspirin on Major Bleeding and Cardiovascular Events in Patients With Acute Coronary Syndrome: The TICO Randomized Clinical Trial. Jama. 2020;323(23):2407-16.
125. Kirchhof P, Camm AJ, Goette A, Brandes A, Eckardt L, Elvan A, et al. Early Rhythm-Control Therapy in Patients with Atrial Fibrillation. The New England journal of medicine. 2020;383(14):1305-16.
126. Kirpalani H, Bell EF, Hintz SR, Tan S, Schmidt B, Chaudhary AS, et al. Higher or Lower Hemoglobin Transfusion Thresholds for Preterm Infants. The New England journal of medicine. 2020;383(27):2639-51.
127. Klein AL, Imazio M, Cremer P, Brucato A, Abbate A, Fang F, et al. Phase 3 Trial of Interleukin-1 Trap Rilonacept in Recurrent Pericarditis. The New England journal of medicine. 2021;384(1):31-41.
128. Koblan KS, Kent J, Hopkins SC, Krystal JH, Cheng H, Goldman R, et al. A Non-D2-Receptor-Binding Drug for the Treatment of Schizophrenia. The New England journal of medicine. 2020;382(16):1497-506.

129. Kotecha D, Bunting KV, Gill SK, Mehta S, Stanbury M, Jones JC, et al. Effect of Digoxin vs Bisoprolol for Heart Rate Control in Atrial Fibrillation on Patient-Reported Quality of Life: The RATE-AF Randomized Clinical Trial. Jama. 2020;324(24):2497-508.
130. Kozal M, Aberg J, Pialoux G, Cahn P, Thompson M, Molina JM, et al. Fostemsavir in Adults with Multidrug-Resistant HIV-1 Infection. The New England journal of medicine. 2020;382(13):1232-43.
131. Kuppermann M, Kaimal AJ, Blat C, Gonzalez J, Thiet MP, Bermingham Y, et al. Effect of a Patient-Centered Decision Support Tool on Rates of Trial of Labor After Previous Cesarean Delivery: The PROCEED Randomized Clinical Trial. Jama. 2020;323(21):2151-9.
132. Kwambai TK, Dhabangi A, Idro R, Opoka R, Watson V, Kariuki S, et al. Malaria Chemoprevention in the Postdischarge Management of Severe Anemia. The New England journal of medicine. 2020;383(23):2242-54.
133. Lacasse Y, Sériès F, Corbeil F, Baltzan M, Paradis B, Simão P, et al. Randomized Trial of Nocturnal Oxygen in Chronic Obstructive Pulmonary Disease. The New England journal of medicine. 2020;383(12):1129-38.
134. Laffel LM, Kanapka LG, Beck RW, Bergamo K, Clements MA, Criego A, et al. Effect of Continuous Glucose Monitoring on Glycemic Control in Adolescents and Young Adults With Type 1 Diabetes: A Randomized Clinical Trial. Jama. 2020;323(23):2388-96.
135. Lamontagne F, Richards-Belle A, Thomas K, Harrison DA, Sadique MZ, Grieve RD, et al. Effect of Reduced Exposure to Vasopressors on 90-Day Mortality in Older Critically Ill Patients With Vasodilatory Hypotension: A Randomized Clinical Trial. Jama. 2020;323(10):938-49.
136. Lau JYW, Yu Y, Tang RSY, Chan HCH, Yip HC, Chan SM, et al. Timing of Endoscopy for Acute Upper Gastrointestinal Bleeding. The New England journal of medicine. 2020;382(14):1299-308.
137. Lebwohl MG, Papp KA, Stein Gold L, Gooderham MJ, Kircik LH, Draelos ZD, et al. Trial of Roflumilast Cream for Chronic Plaque Psoriasis. The New England journal of medicine. 2020;383(3):229-39.
138. Lenze EJ, Mattar C, Zorumski CF, Stevens A, Schweiger J, Nicol GE, et al. Fluvoxamine vs Placebo and Clinical Deterioration in Outpatients With Symptomatic COVID-19: A Randomized Clinical Trial. Jama. 2020;324(22):2292-300.
139. Lévy Y, Lelièvre JD, Assoumou L, Aznar E, Pulido F, Tambussi G, et al. Addition of Maraviroc Versus Placebo to Standard Antiretroviral Therapy for Initial Treatment of Advanced HIV Infection: A Randomized Trial. Annals of internal medicine. 2020;172(5):297-305.
140. Li L, Zhang W, Hu Y, Tong X, Zheng S, Yang J, et al. Effect of Convalescent Plasma Therapy on Time to Clinical Improvement in Patients With Severe and Life-threatening COVID-19: A Randomized Clinical Trial. Jama. 2020;324(5):460-70.
141. Lomivorotov V, Kornilov I, Boboshko V, Shmyrev V, Bondarenko I, Soynov I, et al. Effect of Intraoperative Dexamethasone on Major Complications and Mortality Among Infants Undergoing Cardiac Surgery: The DECISION Randomized Clinical Trial. Jama. 2020;323(24):2485-92.
142. Lotery A, Sivaprasad S, O'Connell A, Harris RA, Culliford L, Ellis L, et al. Eplerenone for chronic central serous chorioretinopathy in patients with active, previously untreated disease for more than 4 months (VICI): a randomised, double-blind, placebo-controlled trial. Lancet (London, England). 2020;395(10220):294-303.
143. MacKay S, Carney AS, Catcheside PG, Chai-Coetzer CL, Chia M, Cistulli PA, et al. Effect of Multilevel Upper Airway Surgery vs Medical Management on the Apnea-Hypopnea Index and Patient-Reported Daytime Sleepiness Among Patients With Moderate or Severe Obstructive Sleep Apnea: The SAMS Randomized Clinical Trial. Jama. 2020;324(12):1168-79.
144. Mackle D, Bellomo R, Bailey M, Beasley R, Deane A, Eastwood G, et al. Conservative Oxygen Therapy during Mechanical Ventilation in the ICU. The New England journal of medicine. 2020;382(11):989-98.

145. Madhi SA, Polack FP, Piedra PA, Munoz FM, Trenholme AA, Simões EAF, et al. Respiratory Syncytial Virus Vaccination during Pregnancy and Effects in Infants. The New England journal of medicine. 2020;383(5):426-39.
146. Manyonda I, Belli AM, Lumsden MA, Moss J, McKinnon W, Middleton LJ, et al. Uterine-Artery Embolization or Myomectomy for Uterine Fibroids. The New England journal of medicine. 2020;383(5):440-51.
147. Marc I, Piedboeuf B, Lacaze-Masmonteil T, Fraser W, Mâsse B, Mohamed I, et al. Effect of Maternal Docosahexaenoic Acid Supplementation on Bronchopulmonary Dysplasia-Free Survival in Breastfed Preterm Infants: A Randomized Clinical Trial. Jama. 2020;324(2):157-67.
148. Maron DJ, Hochman JS, Reynolds HR, Bangalore S, O'Brien SM, Boden WE, et al. Initial Invasive or Conservative Strategy for Stable Coronary Disease. The New England journal of medicine. 2020;382(15):1395-407.
149. Martínez-Fernández R, Máñez-Miró JU, Rodríguez-Rojas R, Del Álamo M, Shah BB, Hernández-Fernández F, et al. Randomized Trial of Focused Ultrasound Subthalamotomy for Parkinson's Disease. The New England journal of medicine. 2020;383(26):2501-13.
150. Martins SO, Mont'Alverne F, Rebello LC, Abud DG, Silva GS, Lima FO, et al. Thrombectomy for Stroke in the Public Health Care System of Brazil. The New England journal of medicine. 2020;382(24):2316-26.
151. Maskew M, Brennan AT, Fox MP, Vezi L, Venter WDF, Ehrenkranz P, et al. A clinical algorithm for same-day HIV treatment initiation in settings with high TB symptom prevalence in South Africa: The SLATE II individually randomized clinical trial. PLoS medicine. 2020;17(8):e1003226.
152. McInnes IB, Behrens F, Mease PJ, Kavanaugh A, Ritchlin C, Nash P, et al. Secukinumab versus adalimumab for treatment of active psoriatic arthritis (EXCEED): a double-blind, parallel-group, randomised, active-controlled, phase 3b trial. Lancet (London, England). 2020;395(10235):1496-505.
153. Mease PJ, Rahman P, Gottlieb AB, Kollmeier AP, Hsia EC, Xu XL, et al. Guselkumab in biologic-naive patients with active psoriatic arthritis (DISCOVER-2): a double-blind, randomised, placebo-controlled phase 3 trial. Lancet (London, England). 2020;395(10230):1126-36.
154. Milstone AM, Voskertchian A, Koontz DW, Khamash DF, Ross T, Aucott SW, et al. Effect of Treating Parents Colonized With Staphylococcus aureus on Transmission to Neonates in the Intensive Care Unit: A Randomized Clinical Trial. Jama. 2020;323(4):319-28.
155. Minard-Colin V, Aupérin A, Pillon M, Burke GAA, Barkauskas DA, Wheatley K, et al. Rituximab for High-Risk, Mature B-Cell Non-Hodgkin's Lymphoma in Children. The New England journal of medicine. 2020;382(23):2207-19.
156. Mittendorf EA, Zhang H, Barrios CH, Saji S, Jung KH, Hegg R, et al. Neoadjuvant atezolizumab in combination with sequential nab-paclitaxel and anthracycline-based chemotherapy versus placebo and chemotherapy in patients with early-stage triple-negative breast cancer (IMpassion031): a randomised, double-blind, phase 3 trial. Lancet (London, England). 2020;396(10257):1090-100.
157. Morand EF, Furie R, Tanaka Y, Bruce IN, Askanase AD, Richez C, et al. Trial of Anifrolumab in Active Systemic Lupus Erythematosus. The New England journal of medicine. 2020;382(3):211-21.
158. Moskowitz A, Huang DT, Hou PC, Gong J, Doshi PB, Grossestreuer AV, et al. Effect of Ascorbic Acid, Corticosteroids, and Thiamine on Organ Injury in Septic Shock: The ACTS Randomized Clinical Trial. Jama. 2020;324(7):642-50.
159. Murray LK, Kane JC, Glass N, Skavenski van Wyk S, Melendez F, Paul R, et al. Effectiveness of the Common Elements Treatment Approach (CETA) in reducing intimate partner violence and hazardous alcohol use in Zambia (VATU): A randomized controlled trial. PLoS medicine. 2020;17(4):e1003056.
160. Murthy RK, Loi S, Okines A, Paplomata E, Hamilton E, Hurvitz SA, et al. Tucatinib, Trastuzumab, and Capecitabine for HER2-Positive Metastatic Breast Cancer. The New England journal of medicine. 2020;382(7):597-609.

161. Newsome PN, Buchholtz K, Cusi K, Linder M, Okanoue T, Ratziu V, et al. A Placebo-Controlled Trial of Subcutaneous Semaglutide in Nonalcoholic Steatohepatitis. The New England journal of medicine. 2020.
162. Nicholls SJ, Lincoff AM, Garcia M, Bash D, Ballantyne CM, Barter PJ, et al. Effect of High-Dose Omega-3 Fatty Acids vs Corn Oil on Major Adverse Cardiovascular Events in Patients at High Cardiovascular Risk: The STRENGTH Randomized Clinical Trial. Jama. 2020;324(22):2268-80.
163. Nidorf SM, Fiolet ATL, Mosterd A, Eikelboom JW, Schut A, Opstal TSJ, et al. Colchicine in Patients with Chronic Coronary Disease. The New England journal of medicine. 2020;383(19):1838-47.
164. Nijenhuis VJ, Brouwer J, Delewi R, Hermanides RS, Holvoet W, Dubois CLF, et al. Anticoagulation with or without Clopidogrel after Transcatheter Aortic-Valve Implantation. The New England journal of medicine. 2020;382(18):1696-707.
165. Nowak H, Zech N, Asmussen S, Rahmel T, Tryba M, Oprea G, et al. Effect of therapeutic suggestions during general anaesthesia on postoperative pain and opioid use: multicentre randomised controlled trial. BMJ (Clinical research ed). 2020;371:m4284.
166. Okumura K, Akao M, Yoshida T, Kawata M, Okazaki O, Akashi S, et al. Low-Dose Edoxaban in Very Elderly Patients with Atrial Fibrillation. The New England journal of medicine. 2020;383(18):1735-45.
167. Olavarria OA, Bernardi K, Shah SK, Wilson TD, Wei S, Pedroza C, et al. Robotic versus laparoscopic ventral hernia repair: multicenter, blinded randomized controlled trial. BMJ (Clinical research ed). 2020;370:m2457.
168. Olivotto I, Oreziak A, Barriales-Villa R, Abraham TP, Masri A, Garcia-Pavia P, et al. Mavacamten for treatment of symptomatic obstructive hypertrophic cardiomyopathy (EXPLORER-HCM): a randomised, double-blind, placebo-controlled, phase 3 trial. Lancet (London, England). 2020;396(10253):759-69.
169. Olsen HT, Nedergaard HK, Strøm T, Oxlund J, Wian KA, Ytrebø LM, et al. Nonsedation or Light Sedation in Critically Ill, Mechanically Ventilated Patients. The New England journal of medicine. 2020;382(12):1103-11.
170. Ono S, Kawada K, Dohi O, Kitamura S, Koike T, Hori S, et al. Linked Color Imaging Focused on Neoplasm Detection in the Upper Gastrointestinal Tract : A Randomized Trial. Annals of internal medicine. 2021;174(1):18-24.
171. Packer M, Anker SD, Butler J, Filippatos G, Pocock SJ, Carson P, et al. Cardiovascular and Renal Outcomes with Empagliflozin in Heart Failure. The New England journal of medicine. 2020;383(15):1413-24.
172. Paganoni S, Macklin EA, Hendrix S, Berry JD, Elliott MA, Maiser S, et al. Trial of Sodium Phenylbutyrate-Taurursodiol for Amyotrophic Lateral Sclerosis. The New England journal of medicine. 2020;383(10):919-30.
173. Park ER, Perez GK, Regan S, Muzikansky A, Levy DE, Temel JS, et al. Effect of Sustained Smoking Cessation Counseling and Provision of Medication vs Shorter-term Counseling and Medication Advice on Smoking Abstinence in Patients Recently Diagnosed With Cancer: A Randomized Clinical Trial. Jama. 2020;324(14):1406-18.
174. Parsons JK, Zahrieh D, Mohler JL, Paskett E, Hansel DE, Kibel AS, et al. Effect of a Behavioral Intervention to Increase Vegetable Consumption on Cancer Progression Among Men With Early-Stage Prostate Cancer: The MEAL Randomized Clinical Trial. Jama. 2020;323(2):140-8.
175. Pereira NL, Farkouh ME, So D, Lennon R, Geller N, Mathew V, et al. Effect of Genotype-Guided Oral P2Y12 Inhibitor Selection vs Conventional Clopidogrel Therapy on Ischemic Outcomes After Percutaneous Coronary Intervention: The TAILOR-PCI Randomized Clinical Trial. Jama. 2020;324(8):761-71.
176. Pleguezuelos O, Dille J, de Groen S, Oftung F, Niesters HGM, Islam MA, et al. Immunogenicity, Safety, and Efficacy of a Standalone Universal Influenza Vaccine, FLU-v, in Healthy Adults: A Randomized Clinical Trial. Annals of internal medicine. 2020;172(7):453-62.

177. Polack FP, Thomas SJ, Kitchin N, Absalon J, Gurtman A, Lockhart S, et al. Safety and Efficacy of the BNT162b2 mRNA Covid-19 Vaccine. The New England journal of medicine. 2020;383(27):2603-15.
178. Ponikowski P, Kirwan BA, Anker SD, McDonagh T, Dorobantu M, Drozdz J, et al. Ferric carboxymaltose for iron deficiency at discharge after acute heart failure: a multicentre, double-blind, randomised, controlled trial. Lancet (London, England). 2020;396(10266):1895-904.
179. Post R, Germans MR, Tjerkstra MA, Vergouwen MDI, Jellema K, Koot RW, et al. Ultra-early tranexamic acid after subarachnoid haemorrhage (ULTRA): a randomised controlled trial. Lancet (London, England). 2021;397(10269):112-8.
180. Powles T, Park SH, Voog E, Caserta C, Valderrama BP, Gurney H, et al. Avelumab Maintenance Therapy for Advanced or Metastatic Urothelial Carcinoma. The New England journal of medicine. 2020;383(13):1218-30.
181. Pratley RE, Kanapka LG, Rickels MR, Ahmann A, Aleppo G, Beck R, et al. Effect of Continuous Glucose Monitoring on Hypoglycemia in Older Adults With Type 1 Diabetes: A Randomized Clinical Trial. Jama. 2020;323(23):2397-406.
182. Quattrin T, Haller MJ, Steck AK, Felner EI, Li Y, Xia Y, et al. Golimumab and Beta-Cell Function in Youth with New-Onset Type 1 Diabetes. The New England journal of medicine. 2020;383(21):2007-17.
183. Raal FJ, Kallend D, Ray KK, Turner T, Koenig W, Wright RS, et al. Inclisiran for the Treatment of Heterozygous Familial Hypercholesterolemia. The New England journal of medicine. 2020;382(16):1520-30.
184. Raal FJ, Rosenson RS, Reeskamp LF, Hovingh GK, Kastelein JJP, Rubba P, et al. Evinacumab for Homozygous Familial Hypercholesterolemia. The New England journal of medicine. 2020;383(8):711-20.
185. Rabe KF, Martinez FJ, Ferguson GT, Wang C, Singh D, Wedzicha JA, et al. Triple Inhaled Therapy at Two Glucocorticoid Doses in Moderate-to-Very-Severe COPD. The New England journal of medicine. 2020;383(1):35-48.
186. Rämö L, Sumrein BO, Lepola V, Lähdeoja T, Ranstam J, Paavola M, et al. Effect of Surgery vs Functional Bracing on Functional Outcome Among Patients With Closed Displaced Humeral Shaft Fractures: The FISH Randomized Clinical Trial. Jama. 2020;323(18):1792-801.
187. Rangan A, Brealey SD, Keding A, Corbacho B, Northgraves M, Kottam L, et al. Management of adults with primary frozen shoulder in secondary care (UK FROST): a multicentre, pragmatic, three-arm, superiority randomised clinical trial. Lancet (London, England). 2020;396(10256):977-89.
188. Ranieri VM, Pettilä V, Karvonen MK, Jalkanen J, Nightingale P, Brealey D, et al. Effect of Intravenous Interferon β-1a on Death and Days Free From Mechanical Ventilation Among Patients With Moderate to Severe Acute Respiratory Distress Syndrome: A Randomized Clinical Trial. Jama. 2020;323(8):725-33.
189. Ray KK, Nicholls SJ, Buhr KA, Ginsberg HN, Johansson JO, Kalantar-Zadeh K, et al. Effect of Apabetalone Added to Standard Therapy on Major Adverse Cardiovascular Events in Patients With Recent Acute Coronary Syndrome and Type 2 Diabetes: A Randomized Clinical Trial. Jama. 2020;323(16):1565-73.
190. Reichenbach S, Felson DT, Hincapié CA, Heldner S, Bütikofer L, Lenz A, et al. Effect of Biomechanical Footwear on Knee Pain in People With Knee Osteoarthritis: The BIOTOK Randomized Clinical Trial. Jama. 2020;323(18):1802-12.
191. Richards T, Baikady RR, Clevenger B, Butcher A, Abeysiri S, Chau M, et al. Preoperative intravenous iron to treat anaemia before major abdominal surgery (PREVENTT): a randomised, double-blind, controlled trial. Lancet (London, England). 2020;396(10259):1353-61.
192. Ritchlin CT, Kavanaugh A, Merola JF, Schett G, Scher JU, Warren RB, et al. Bimekizumab in patients with active psoriatic arthritis: results from a 48-week, randomised, double-blind, placebo-controlled, dose-ranging phase 2b trial. Lancet (London, England). 2020;395(10222):427-40.

193. Rosenson RS, Burgess LJ, Ebenbichler CF, Baum SJ, Stroes ESG, Ali S, et al. Evinacumab in Patients with Refractory Hypercholesterolemia. The New England journal of medicine. 2020;383(24):2307-19.
194. Rosenstock J, Bajaj HS, Janež A, Silver R, Begtrup K, Hansen MV, et al. Once-Weekly Insulin for Type 2 Diabetes without Previous Insulin Treatment. The New England journal of medicine. 2020;383(22):2107-16.
195. Rosmarin D, Pandya AG, Lebwohl M, Grimes P, Hamzavi I, Gottlieb AB, et al. Ruxolitinib cream for treatment of vitiligo: a randomised, controlled, phase 2 trial. Lancet (London, England). 2020;396(10244):110-20.
196. Rowell SE, Meier EN, McKnight B, Kannas D, May S, Sheehan K, et al. Effect of Out-of-Hospital Tranexamic Acid vs Placebo on 6-Month Functional Neurologic Outcomes in Patients With Moderate or Severe Traumatic Brain Injury. Jama. 2020;324(10):961-74.
197. Salama C, Han J, Yau L, Reiss WG, Kramer B, Neidhart JD, et al. Tocilizumab in Patients Hospitalized with Covid-19 Pneumonia. The New England journal of medicine. 2021;384(1):20-30.
198. Santos RD, Ruzza A, Hovingh GK, Wiegman A, Mach F, Kurtz CE, et al. Evolocumab in Pediatric Heterozygous Familial Hypercholesterolemia. The New England journal of medicine. 2020;383(14):1317-27.
199. Savarirayan R, Tofts L, Irving M, Wilcox W, Bacino CA, Hoover-Fong J, et al. Once-daily, subcutaneous vosoritide therapy in children with achondroplasia: a randomised, double-blind, phase 3, placebo-controlled, multicentre trial. Lancet (London, England). 2020;396(10252):684-92.
200. Schepers NJ, Hallensleben NDL, Besselink MG, Anten MGF, Bollen TL, da Costa DW, et al. Urgent endoscopic retrograde cholangiopancreatography with sphincterotomy versus conservative treatment in predicted severe acute gallstone pancreatitis (APEC): a multicentre randomised controlled trial. Lancet (London, England). 2020;396(10245):167-76.
201. Schisterman EF, Sjaarda LA, Clemons T, Carrell DT, Perkins NJ, Johnstone E, et al. Effect of Folic Acid and Zinc Supplementation in Men on Semen Quality and Live Birth Among Couples Undergoing Infertility Treatment: A Randomized Clinical Trial. Jama. 2020;323(1):35-48.
202. Schuh S, Sweeney J, Rumantir M, Coates AL, Willan AR, Stephens D, et al. Effect of Nebulized Magnesium vs Placebo Added to Albuterol on Hospitalization Among Children With Refractory Acute Asthma Treated in the Emergency Department: A Randomized Clinical Trial. Jama. 2020;324(20):2038-47.
203. Schweitzer C, Brezin A, Cochener B, Monnet D, Germain C, Roseng S, et al. Femtosecond laser-assisted versus phacoemulsification cataract surgery (FEMCAT): a multicentre participant-masked randomised superiority and cost-effectiveness trial. Lancet (London, England). 2020;395(10219):212-24.
204. Self WH, Semler MW, Leither LM, Casey JD, Angus DC, Brower RG, et al. Effect of Hydroxychloroquine on Clinical Status at 14 Days in Hospitalized Patients With COVID-19: A Randomized Clinical Trial. Jama. 2020;324(21):2165-76.
205. Shahar-Nissan K, Pardo J, Peled O, Krause I, Bilavsky E, Wiznitzer A, et al. Valaciclovir to prevent vertical transmission of cytomegalovirus after maternal primary infection during pregnancy: a randomised, double-blind, placebo-controlled trial. Lancet (London, England). 2020;396(10253):779-85.
206. Sharman JP, Egyed M, Jurczak W, Skarbnik A, Pagel JM, Flinn IW, et al. Acalabrutinib with or without obinutuzumab versus chlorambucil and obinutuzmab for treatment-naive chronic lymphocytic leukaemia (ELEVATE TN): a randomised, controlled, phase 3 trial. Lancet (London, England). 2020;395(10232):1278-91.
207. Shitara K, Bang YJ, Iwasa S, Sugimoto N, Ryu MH, Sakai D, et al. Trastuzumab Deruxtecan in Previously Treated HER2-Positive Gastric Cancer. The New England journal of medicine. 2020;382(25):2419-30.

208. Shore ND, Saad F, Cookson MS, George DJ, Saltzstein DR, Tutrone R, et al. Oral Relugolix for Androgen-Deprivation Therapy in Advanced Prostate Cancer. The New England journal of medicine. 2020;382(23):2187-96.
209. Silvain J, Lattuca B, Beygui F, Rangé G, Motovska Z, Dillinger JG, et al. Ticagrelor versus clopidogrel in elective percutaneous coronary intervention (ALPHEUS): a randomised, open-label, phase 3b trial. Lancet (London, England). 2020;396(10264):1737-44.
210. Simonovich VA, Burgos Pratx LD, Scibona P, Beruto MV, Vallone MG, Vázquez C, et al. A Randomized Trial of Convalescent Plasma in Covid-19 Severe Pneumonia. The New England journal of medicine. 2020.
211. Simpson EL, Sinclair R, Forman S, Wollenberg A, Aschoff R, Cork M, et al. Efficacy and safety of abrocitinib in adults and adolescents with moderate-to-severe atopic dermatitis (JADE MONO-1): a multicentre, double-blind, randomised, placebo-controlled, phase 3 trial. Lancet (London, England). 2020;396(10246):255-66.
212. Singh N, Winston DJ, Razonable RR, Lyon GM, Silveira FP, Wagener MM, et al. Effect of Preemptive Therapy vs Antiviral Prophylaxis on Cytomegalovirus Disease in Seronegative Liver Transplant Recipients With Seropositive Donors: A Randomized Clinical Trial. Jama. 2020;323(14):1378-87.
213. Skipper CP, Pastick KA, Engen NW, Bangdiwala AS, Abassi M, Lofgren SM, et al. Hydroxychloroquine in Nonhospitalized Adults With Early COVID-19 : A Randomized Trial. Annals of internal medicine. 2020;173(8):623-31.
214. Spinner CD, Gottlieb RL, Criner GJ, Arribas López JR, Cattelan AM, Soriano Viladomiu A, et al. Effect of Remdesivir vs Standard Care on Clinical Status at 11 Days in Patients With Moderate COVID-19: A Randomized Clinical Trial. Jama. 2020;324(11):1048-57.
215. Ständer S, Yosipovitch G, Legat FJ, Lacour JP, Paul C, Narbutt J, et al. Trial of Nemolizumab in Moderate-to-Severe Prurigo Nodularis. The New England journal of medicine. 2020;382(8):706-16.
216. Steinberg JS, Shabanov V, Ponomarev D, Losik D, Ivanickiy E, Kropotkin E, et al. Effect of Renal Denervation and Catheter Ablation vs Catheter Ablation Alone on Atrial Fibrillation Recurrence Among Patients With Paroxysmal Atrial Fibrillation and Hypertension: The ERADICATE-AF Randomized Clinical Trial. Jama. 2020;323(3):248-55.
217. Stensvold D, Viken H, Steinshamn SL, Dalen H, Støylen A, Loennechen JP, et al. Effect of exercise training for five years on all cause mortality in older adults-the Generation 100 study: randomised controlled trial. BMJ (Clinical research ed). 2020;371:m3485.
218. Stone JH, Frigault MJ, Serling-Boyd NJ, Fernandes AD, Harvey L, Foulkes AS, et al. Efficacy of Tocilizumab in Patients Hospitalized with Covid-19. The New England journal of medicine. 2020;383(24):2333-44.
219. Stormlund S, Sopa N, Zedeler A, Bogstad J, Prætorius L, Nielsen HS, et al. Freeze-all versus fresh blastocyst transfer strategy during in vitro fertilisation in women with regular menstrual cycles: multicentre randomised controlled trial. BMJ (Clinical research ed). 2020;370:m2519.
220. Strand TA, Ulak M, Hysing M, Ranjitkar S, Kvestad I, Shrestha M, et al. Effects of vitamin B12 supplementation on neurodevelopment and growth in Nepalese Infants: A randomized controlled trial. PLoS medicine. 2020;17(12):e1003430.
221. Tanaka A, Taguchi I, Teragawa H, Ishizaka N, Kanzaki Y, Tomiyama H, et al. Febuxostat does not delay progression of carotid atherosclerosis in patients with asymptomatic hyperuricemia: A randomized, controlled trial. PLoS medicine. 2020;17(4):e1003095.
222. Tang W, Cao Z, Han M, Wang Z, Chen J, Sun W, et al. Hydroxychloroquine in patients with mainly mild to moderate coronavirus disease 2019: open label, randomised controlled trial. BMJ (Clinical research ed). 2020;369:m1849.
223. Tap WD, Wagner AJ, Schöffski P, Martin-Broto J, Krarup-Hansen A, Ganjoo KN, et al. Effect of Doxorubicin Plus Olaratumab vs Doxorubicin Plus Placebo on Survival in Patients With Advanced Soft Tissue Sarcomas: The ANNOUNCE Randomized Clinical Trial. Jama. 2020;323(13):1266-76.

224. Tay AK, Mung HK, Miah MAA, Balasundaram S, Ventevogel P, Badrudduza M, et al. An Integrative Adapt Therapy for common mental health symptoms and adaptive stress amongst Rohingya, Chin, and Kachin refugees living in Malaysia: A randomized controlled trial. PLoS medicine. 2020;17(3):e1003073.
225. Teerlink JR, Diaz R, Felker GM, McMurray JJV, Metra M, Solomon SD, et al. Cardiac Myosin Activation with Omecamtiv Mecarbil in Systolic Heart Failure. The New England journal of medicine. 2021;384(2):105-16.
226. Tomazini BM, Maia IS, Cavalcanti AB, Berwanger O, Rosa RG, Veiga VC, et al. Effect of Dexamethasone on Days Alive and Ventilator-Free in Patients With Moderate or Severe Acute Respiratory Distress Syndrome and COVID-19: The CoDEX Randomized Clinical Trial. Jama. 2020;324(13):1307-16.
227. Tong SYC, Lye DC, Yahav D, Sud A, Robinson JO, Nelson J, et al. Effect of Vancomycin or Daptomycin With vs Without an Antistaphylococcal β-Lactam on Mortality, Bacteremia, Relapse, or Treatment Failure in Patients With MRSA Bacteremia: A Randomized Clinical Trial. Jama. 2020;323(6):527-37.
228. Trapnell BC, Inoue Y, Bonella F, Morgan C, Jouneau S, Bendstrup E, et al. Inhaled Molgramostim Therapy in Autoimmune Pulmonary Alveolar Proteinosis. The New England journal of medicine. 2020;383(17):1635-44.
229. Tricou V, Sáez-Llorens X, Yu D, Rivera L, Jimeno J, Villarreal AC, et al. Safety and immunogenicity of a tetravalent dengue vaccine in children aged 2-17 years: a randomised, placebo-controlled, phase 2 trial. Lancet (London, England). 2020;395(10234):1434-43.
230. Tsimikas S, Karwatowska-Prokopczuk E, Gouni-Berthold I, Tardif JC, Baum SJ, Steinhagen-Thiessen E, et al. Lipoprotein(a) Reduction in Persons with Cardiovascular Disease. The New England journal of medicine. 2020;382(3):244-55.
231. Turan A, Duncan A, Leung S, Karimi N, Fang J, Mao G, et al. Dexmedetomidine for reduction of atrial fibrillation and delirium after cardiac surgery (DECADE): a randomised placebo-controlled trial. Lancet (London, England). 2020;396(10245):177-85.
232. Turan A, Essber H, Saasouh W, Hovsepyan K, Makarova N, Ayad S, et al. Effect of Intravenous Acetaminophen on Postoperative Hypoxemia After Abdominal Surgery: The FACTOR Randomized Clinical Trial. Jama. 2020;324(4):350-8.
233. Tuuli MG, Liu J, Tita ATN, Longo S, Trudell A, Carter EB, et al. Effect of Prophylactic Negative Pressure Wound Therapy vs Standard Wound Dressing on Surgical-Site Infection in Obese Women After Cesarean Delivery: A Randomized Clinical Trial. Jama. 2020;324(12):1180-9.
234. Udelson JE, Lewis GD, Shah SJ, Zile MR, Redfield MM, Burnett J, Jr., et al. Effect of Praliciguat on Peak Rate of Oxygen Consumption in Patients With Heart Failure With Preserved Ejection Fraction: The CAPACITY HFpEF Randomized Clinical Trial. Jama. 2020;324(15):1522-31.
235. Valderrábano M, Peterson LE, Swarup V, Schurmann PA, Makkar A, Doshi RN, et al. Effect of Catheter Ablation With Vein of Marshall Ethanol Infusion vs Catheter Ablation Alone on Persistent Atrial Fibrillation: The VENUS Randomized Clinical Trial. Jama. 2020;324(16):1620-8.
236. van der Pluijm RW, Tripura R, Hoglund RM, Pyae Phyo A, Lek D, Ul Islam A, et al. Triple artemisinin-based combination therapies versus artemisinin-based combination therapies for uncomplicated Plasmodium falciparum malaria: a multicentre, open-label, randomised clinical trial. Lancet (London, England). 2020;395(10233):1345-60.
237. van der Vlist AC, van Oosterom RF, van Veldhoven PLJ, Bierma-Zeinstra SMA, Waarsing JH, Verhaar JAN, et al. Effectiveness of a high volume injection as treatment for chronic Achilles tendinopathy: randomised controlled trial. BMJ (Clinical research ed). 2020;370:m3027.
238. Voskoboinik A, Kalman JM, De Silva A, Nicholls T, Costello B, Nanayakkara S, et al. Alcohol Abstinence in Drinkers with Atrial Fibrillation. The New England journal of medicine. 2020;382(1):20-8.

239. Walline JJ, Walker MK, Mutti DO, Jones-Jordan LA, Sinnott LT, Giannoni AG, et al. Effect of High Add Power, Medium Add Power, or Single-Vision Contact Lenses on Myopia Progression in Children: The BLINK Randomized Clinical Trial. Jama. 2020;324(6):571-80.
240. Walsh M, Merkel PA, Peh CA, Szpirt WM, Puéchal X, Fujimoto S, et al. Plasma Exchange and Glucocorticoids in Severe ANCA-Associated Vasculitis. The New England journal of medicine. 2020;382(7):622-31.
241. Wang Y, Zhang D, Du G, Du R, Zhao J, Jin Y, et al. Remdesivir in adults with severe COVID-19: a randomised, double-blind, placebo-controlled, multicentre trial. Lancet (London, England). 2020;395(10236):1569-78.
242. Wazni OM, Dandamudi G, Sood N, Hoyt R, Tyler J, Durrani S, et al. Cryoballoon Ablation as Initial Therapy for Atrial Fibrillation. The New England journal of medicine. 2021;384(4):316-24.
243. Webb E, Neeman T, Bowden FJ, Gaida J, Mumford V, Bissett B. Compression Therapy to Prevent Recurrent Cellulitis of the Leg. The New England journal of medicine. 2020;383(7):630-9.
244. Wei AH, Döhner H, Pocock C, Montesinos P, Afanasyev B, Dombret H, et al. Oral Azacitidine Maintenance Therapy for Acute Myeloid Leukemia in First Remission. The New England journal of medicine. 2020;383(26):2526-37.
245. Wijnberge M, Geerts BF, Hol L, Lemmers N, Mulder MP, Berge P, et al. Effect of a Machine Learning-Derived Early Warning System for Intraoperative Hypotension vs Standard Care on Depth and Duration of Intraoperative Hypotension During Elective Noncardiac Surgery: The HYPE Randomized Clinical Trial. Jama. 2020;323(11):1052-60.
246. Wilson AM, Clark AB, Cahn T, Chilvers ER, Fraser W, Hammond M, et al. Effect of Co-trimoxazole (Trimethoprim-Sulfamethoxazole) vs Placebo on Death, Lung Transplant, or Hospital Admission in Patients With Moderate and Severe Idiopathic Pulmonary Fibrosis: The EME-TIPAC Randomized Clinical Trial. Jama. 2020;324(22):2282-91.
247. Xia W, Li HCW, Cai W, Song P, Zhou X, Lam KWK, et al. Effectiveness of a video-based smoking cessation intervention focusing on maternal and child health in promoting quitting among expectant fathers in China: A randomized controlled trial. PLoS medicine. 2020;17(9):e1003355.
248. Xu S, Yu L, Luo X, Wang M, Chen G, Zhang Q, et al. Manual acupuncture versus sham acupuncture and usual care for prophylaxis of episodic migraine without aura: multicentre, randomised clinical trial. BMJ (Clinical research ed). 2020;368:m697.
249. Yang JW, Wang LQ, Zou X, Yan SY, Wang Y, Zhao JJ, et al. Effect of Acupuncture for Postprandial Distress Syndrome: A Randomized Clinical Trial. Annals of internal medicine. 2020;172(12):777-85.
250. Yannopoulos D, Bartos J, Raveendran G, Walser E, Connett J, Murray TA, et al. Advanced reperfusion strategies for patients with out-of-hospital cardiac arrest and refractory ventricular fibrillation (ARREST): a phase 2, single centre, open-label, randomised controlled trial. Lancet (London, England). 2020;396(10265):1807-16.
251. Yusuf S, Joseph P, Dans A, Gao P, Teo K, Xavier D, et al. Polypill with or without Aspirin in Persons without Cardiovascular Disease. The New England journal of medicine. 2021;384(3):216-28.
252. Zarbock A, Küllmar M, Kindgen-Milles D, Wempe C, Gerss J, Brandenburger T, et al. Effect of Regional Citrate Anticoagulation vs Systemic Heparin Anticoagulation During Continuous Kidney Replacement Therapy on Dialysis Filter Life Span and Mortality Among Critically Ill Patients With Acute Kidney Injury: A Randomized Clinical Trial. Jama. 2020;324(16):1629-39.
253. Zeiser R, von Bubnoff N, Butler J, Mohty M, Niederwieser D, Or R, et al. Ruxolitinib for Glucocorticoid-Refractory Acute Graft-versus-Host Disease. The New England journal of medicine. 2020;382(19):1800-10.
254. Zhang C, Koniak-Griffin D, Qian HZ, Goldsamt LA, Wang H, Brecht ML, et al. Impact of providing free HIV self-testing kits on frequency of testing among men who have sex with men and their sexual partners in China: A randomized controlled trial. PLoS medicine. 2020;17(10):e1003365.
255. Zhu FC, Guan XH, Li YH, Huang JY, Jiang T, Hou LH, et al. Immunogenicity and safety of a recombinant adenovirus type-5-vectored COVID-19 vaccine in healthy adults aged 18 years or older:

a randomised, double-blind, placebo-controlled, phase 2 trial. Lancet (London, England). 2020;396(10249):479-88.

# References

1. International Council for Harmonisation of Technical Requirements for Pharmaceuticals for Human Use. *Addendum on Estimands and Sensitivity Analysis in Clinical Trials to the Guideline on Statistical Principles for Clinical Trials*. 20 November 2019 [cited 2020 4th April 2020].
2. Dodd, L.E., et al., *Endpoints for randomised controlled clinical trials for COVID-19 treatments.* Clinical Trials, 2020. **17**(5): p. 472-482.
3. Król, A., et al., *Improving the evaluation of COPD exacerbation treatment effects by accounting for early treatment discontinuations: a post-hoc analysis of randomised clinical trials.* Respiratory research, 2020. **21**(1): p. 158-158.
